# *Plasmodium falciparum* Nucleosomes Exhibit Reduced Stability and Lost Sequence Dependent Nucleosome Positioning

**DOI:** 10.1371/journal.ppat.1006080

**Published:** 2016-12-29

**Authors:** Elisabeth Silberhorn, Uwe Schwartz, Patrick Löffler, Samuel Schmitz, Anne Symelka, Tania de Koning-Ward, Rainer Merkl, Gernot Längst

**Affiliations:** 1 Biochemistry III; Biochemistry Centre Regensburg (BCR), University of Regensburg, Regensburg, Germany; 2 Biochemistry II; Biochemistry Centre Regensburg (BCR), University of Regensburg, Regensburg, Germany; 3 School of Medicine, Deakin University, Waurn Ponds, Australia; Weill Medical College of Cornell University, UNITED STATES

## Abstract

The packaging and organization of genomic DNA into chromatin represents an additional regulatory layer of gene expression, with specific nucleosome positions that restrict the accessibility of regulatory DNA elements. The mechanisms that position nucleosomes *in vivo* are thought to depend on the biophysical properties of the histones, sequence patterns, like phased di-nucleotide repeats and the architecture of the histone octamer that folds DNA in 1.65 tight turns. Comparative studies of human and *P*. *falciparum* histones reveal that the latter have a strongly reduced ability to recognize internal sequence dependent nucleosome positioning signals. In contrast, the nucleosomes are positioned by AT-repeat sequences flanking nucleosomes *in vivo and in vitro*. Further, the strong sequence variations in the plasmodium histones, compared to other mammalian histones, do not present adaptations to its AT-rich genome. Human and parasite histones bind with higher affinity to GC-rich DNA and with lower affinity to AT-rich DNA. However, the plasmodium nucleosomes are overall less stable, with increased temperature induced mobility, decreased salt stability of the histones H2A and H2B and considerable reduced binding affinity to GC-rich DNA, as compared with the human nucleosomes. In addition, we show that plasmodium histone octamers form the shortest known nucleosome repeat length (155bp) *in vitro* and *in vivo*. Our data suggest that the biochemical properties of the parasite histones are distinct from the typical characteristics of other eukaryotic histones and these properties reflect the increased accessibility of the *P*. *falciparum* genome.

## Author Summary

Nucleosomes are not positioned randomly on DNA but on preferential sites with respect to the underlying DNA sequence. Histones belong to the most conserved eukaryotic proteins, as sequence dependent nucleosome positioning is an essential regulatory feature of nucleosomes, determining the accessibility of regulatory factors to DNA. We determined the biochemical properties of plasmodium histones and show that they are distinct from human forms, explaining the accessible chromatin structure of *P*. *falciparum*. Amino acid exchanges in the histones do not present an adaption to the AT-rich genome, but rather reduce the binding affinity to GC-rich DNA sequences, resulting in rather unstable nucleosomes with labile H2A and H2B, requiring extra-nucleosomal positioning signals to keep them on place. Plasmodium chromatin exhibits the shortest nucleosome spacing known to date potentially inhibiting the formation of higher order structures and maintaining chromatin accessible.

## Introduction

The human malaria parasite, *Plasmodium falciparum*, yearly responsible for an estimated 600,000 deaths (WHO Report 2014), has the AT-richest genome sequenced to date. The AT-content averages 80.6% genome wide, but reaches up to 90% in introns and intergenic regions [1]. *P*. *falciparum* shows a complex life cycle in two hosts, exhibiting dramatic changes in the gene expression program. At least 60% of the genome is transcriptionally active during erythrocytic development with gene expression being activated in form of a cascade and tightly regulated during developmental stage transition [2,3].

Like other eukaryotic genomes, the plasmodium forms nucleosomes, possesses chromatin modifiers, chromatin remodeling activities and potentially active DNA methyltransferases [4]. The nucleosome core is composed of 147bp of DNA wrapped 1.65 turns around the histone octamer, consisting of two copies of each H2A, H2B, H3, and H4 [5]. In organizing the nucleosome, H3 homo-dimerizes using the C-terminal ends and heterodimerizes with H4 to form a H3–H4 tetramer [6]. Histone dimers composed either of H2A and H2B, or H3 and H4 organize each 30bp of DNA. Two H3-H4 dimers bind to the central 60bp of nucleosomal DNA and each H2A-H2B dimer organizes 30bp towards the end of the particle [5]. DNA binding and distortion is brought about by the interaction of the histones with the minor groove of the DNA at 14 independent DNA binding regions, termed super helix locations (SHL). Almost 400 direct and indirect histone-DNA interactions render the nucleosome one of the most stable protein-DNA complexes under physiological conditions [5].

The observation that specific DNA sequences favor the formation of nucleosomes *in vitro* and *in vivo*, correlates well with the important role of positioned nucleosomes in organizing the chromatin landscape to regulate gene expression [7–9]. Also for plasmodium it was shown that proper promoter functioning and regulation of var gene expression requires the presence of positioned nucleosomes [10–13]. High-throughput sequencing analyses of nucleosome positions and chromatin structure analyses in different life cycle stages correlate changes in chromatin structure with the regulation of gene expression [14–16]. Interestingly, the chromatin structure of *P*. *falciparum* is distinct from other eukaryotes in that the genome is surprisingly accessible containing poorly positioned nucleosomes [14–17]. These effects are suggested to be related to the extremely high AT-content (81%), generating an inherently inflexible DNA molecule, reducing the potential to form positioned nucleosomes [18,19]. Due to the central role of the histones in the cell, these proteins have been highly conserved throughout eukaryotic evolution [20]. *P*. *falciparum* possesses the most divergent histones in sequence, with identities of only 64%, 67,7%, 92,2% and 92,6% between human and plasmodium H2A, H2B, H4 and H3. This difference may well reflect adaptations for gene regulation and potentially present an adaption to the AT-richness of the genome.

In our study, we show that recombinant plasmodium histones, like human histones, bind poorly to AT-rich DNA. Histone sequence variations result in reduced histone octamer stability and the nucleosomal arrays form the shortest known nucleosome repeat length, measuring only about 155bp. Most interestingly, the octamers lost the capability to recognize intrinsic DNA encoded nucleosome positioning sequences, challenging the current view that DNA structure and di-nucleotide repeats determine translational and rotational nucleosome positioning. Albeit, we observed that the few positioned nucleosomes in *Plasmodium falciparum* are flanked by long AT-repeats in the DNA linker sequences and show that these sequences serve to guide nucleosome positioning. The biochemical properties of the histones mirror the organization of the plasmodium chromatin structure *in vivo* and we show that in contrast to other eukaryotes, long nucleosome flanking, AT-rich sequence elements are required for their positioning.

## Results

### Comparative molecular dynamics simulations of human and parasite nucleosomes

**Fig 1:**
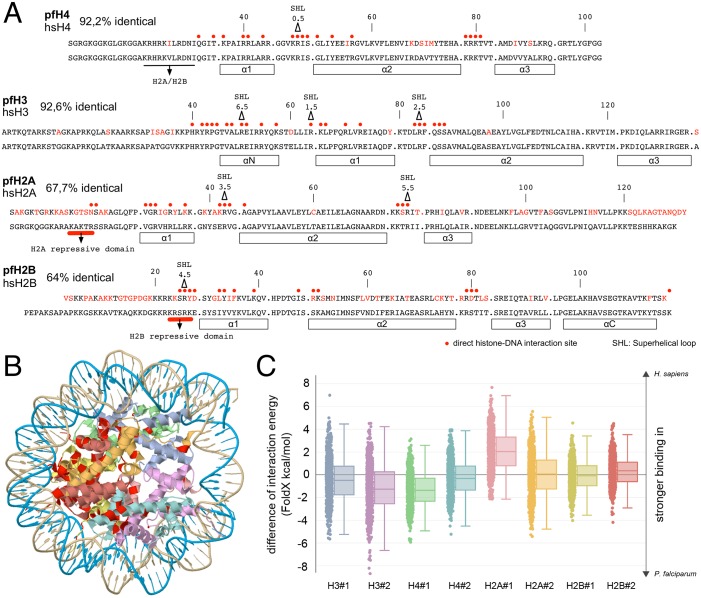
Comparison of human and *P*. *falciparum* histone sequences and their binding energies in the nucleosome core. (*A*) Alignment of the canonical histone sequences, displaying the amino acid changes in *P*. *falciparum* in red color. Major histone-DNA interaction sites are indicated by red circles and the positions of the superhelical loops (SHL) are marked by triangles. (*B*) Crystal structure 1AOI of the human nucleosome, marking the altered amino acids of *P*. *falciparum* in red. (*C*) Differences in FOLDX energies for the histone-DNA interaction. For the *H*. *sapiens* histone cores, the available crystal structure, and for *P*. *falciparum* models of the histone cores were used; see Methods. Energy differences were analyzed for each of the eight individual histones; individual histone copies are indicated by #1 or #2 respectively. Each dot of a scatter plot represents the difference between the median human interaction energy and a snapshot-specific plasmodial energy value. Interactions were further characterized by means of box plots. Whiskers indicate the lowest and the highest datum still within the 1.5 interquartile. The colors correspond to those used in (*B*).

In order to study the functional effects of the distinct *P*. *falciparum* histone sequences, we first analyzed the location of the amino acid differences with respect to nucleosome structure (Fig 1A and 1B). The amino acid differences in H3 and H4 do not affect the sites of direct histone-DNA interaction. H2A and H2B exhibit the majority of differences in their N-terminal tails and the H2A C-terminal tail. In addition clusters of amino acid differences occur in the regions of the superhelix locations (SHL), the regions forming the L1L2 loops, and the α_1_α_2_ DNA-binding motifs [21]. The amino acid differences at the SHL3.5 and 4.5 regions of H2B and H2A affect direct histone-DNA interactions; thereby they could alter nucleosome stability (Fig 1A). In contrast, the variability in the flexible histone tails are suggested not to contribute to complex stability [21].

We used the available nucleosome structure, in the absence of the flexible histone tails (PDB entry 3AFA [22]) to model the strength of the histone-DNA interactions *in silico*. Compared to the full-length sequence, the first 15 and last 13 residues of H2A and the first 27 of H2B, 42 of H3, and 23 residues of H4 were missing. A homology model for the plasmodium nucleosome was built based on the human nucleosome structure (PDB entry 3AFA). The fact that 84% of all amino acid residues in the human nucleosome are identical ensures a high quality 3D-model of the plasmodium nucleosome. Molecular dynamics simulations were performed and DNA-protein interactions for the full complex as well as for individual residues were scored. The histone- and species-specific differences in binding energy were determined by subtracting for each snapshot the score of the plasmodial DNA-histone interaction from the median score calculated for the human DNA-histone interaction (Fig 1C). Only H2A#1 showed a slightly stronger DNA binding in human histones, which we do not consider significant for the following reasons: The reported accuracy of FoldX is 0.46 kcal/mol [23], which is the standard deviation of the difference between ΔΔGs calculated by FoldX and the experimental values. Human H2A sequences differ from plasmodial H2A sequences by 24 residues in the modelled core region, while 14 interacted with DNA in our analysis (see below and S1 Fig). The median difference in H2A#1 energies is 2.03 kcal/mol. Thus, the mean contribution of each mutation is 2.03 kcal/mol / 14 = 0.15 kcal/mol, which is below the reported accuracy. The mean contributions of each mutation are even smaller for the other histones and thus considered as a neutral effect. Therefore, we suggest similar DNA binding for the core regions of all corresponding human and plasmodial histones. For individual residues, π-π stacking, cation-π stacking, contacts, hydrophobic interactions, and hydrogen-bond networks were assessed based on the outcome of YASARA [24]. π-π stacking did not contribute noticeable to DNA-protein interactions and the comparison of all other residue-specific scores did not indicate striking differences (S1 Fig). In summary, the *in silico* modelling suggests a highly similar strength of the interactions between human or plasmodial histone cores and DNA, both in total and on a per residue basis.

### Parasite nucleosomes exhibit reduced stability

**Fig 2:**
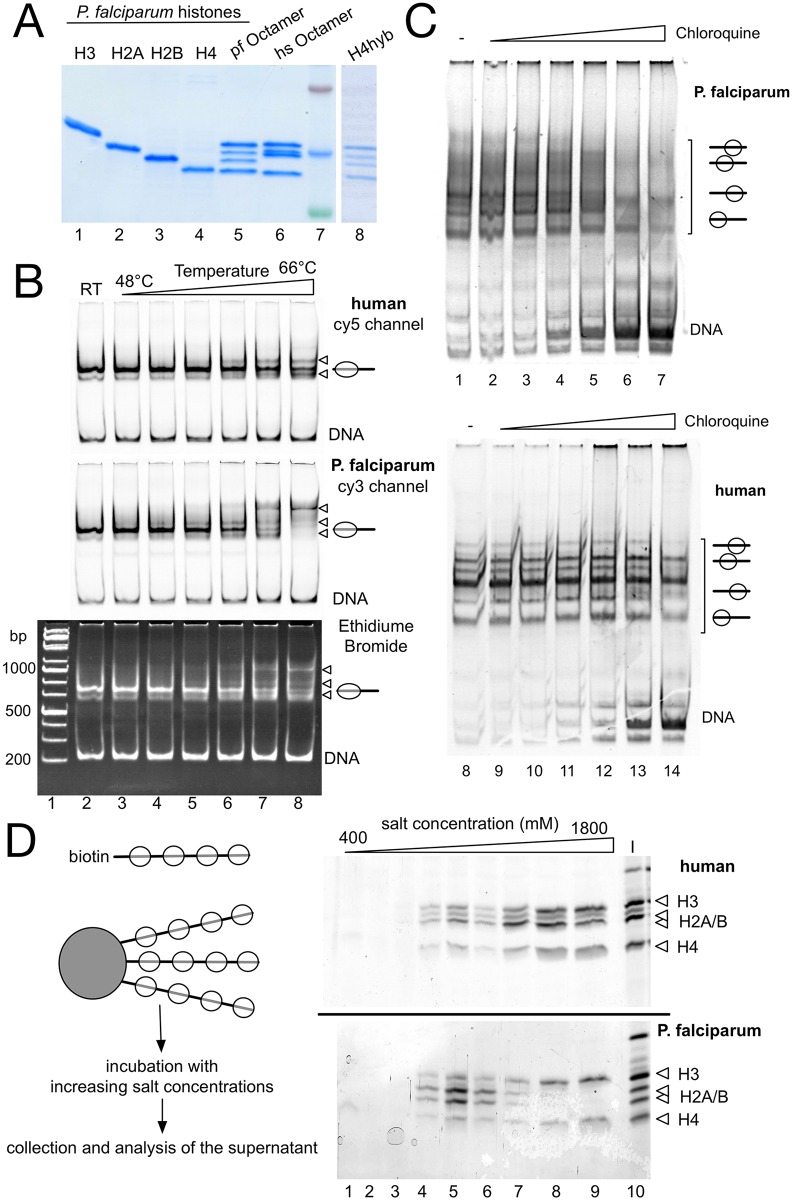
*P*. *falciparum* nucleosomes exhibit reduced heat and salt stabilities. (*A*) Preparation of histone octamers. Histone octamers were reconstituted from appropriate combinations of recombinant parasite and human histones (lanes 1–4), purified by gel filtration, separated by 15% SDS-PAGE and stained with Coomassie blue. The reconstitution H4hyb (lane 8) describes an octamer consisting of the parasite histones H2A, H2B, H3 and the human histone H4. (*B*) Temperature induced nucleosome sliding. *P*. *falciparum* nucleosomes and human nucleosomes were separately reconstituted on Cy3 and Cy5 labelled 601 DNA and then mixed in equimolar ratios. Nucleosomal mixtures were incubated 60 min at room temperature (lane 2) or at the indicated temperatures (lanes 3 to 8). Nucleosome positions were analyzed after the temperature incubation on a native 5% polyacrylamide gel and visualized by fluorescence scanning (upper panel: cy5 labelled human nucleosomes; middle panel: cy3 labelled *P*. *falciparum* nucleosomes) and ethidium bromide staining (lower panel: showing the mixture of human and parasite nucleosomes). The initial nucleosome position and free DNA are indicated and sliding products are marked by triangles. (*C*) Chloroquine stability assay. A 208 bp 601 (GC-rich) and the 210 bp KahrP (AT-rich) DNA fragment were reconstituted either with human (lower panel) or plasmodium octamers (upper panel) into nucleosomes and mixed at equimolar ratio. Nucleosomes were incubated with increasing concentrations of chloroquine (0, 0.05, 0.1, 0.3, 1, 3 and 9 mM), incubated for 10 min at room temperature and then analyzed by native polyacrylamide gel electrophoresis. The DNA and nucleoprotein complexes were visualized by fluorescence imaging staining. The positions of the free DNA and a scheme indicating the different nucleosome positions on the DNA template are given. (*D*) Stepwise salt elution of human and plasmodium histones from DNA. *P*. *falciparum* and human nucleosomes were reconstituted in parallel on linearized, biotinylated DNA and then coupled to magnetic beads. Magnetic beads were incubated stepwise with increasing salt concentrations as indicated and the eluted histones were collected and analyzed by SDS-PAGE and silver staining (lanes 1 to 9). The input fraction is shown in lane 10 and the positions of the histones are indicated. The upper gel shows the results for the human and the lower gel depicts the elution of the parasite histones.

Histones were expressed in bacteria, purified octamers were reconstituted and used for nucleosome assembly (Fig 2). The positions of nucleosomes on DNA can change as a result of thermally induced nucleosome sliding [25,26]. Temperature induced nucleosome sliding exhibits the tendency of moving the nucleosomes to thermodynamically more stable positions, which depend on the DNA sequence [26,27]. We use this method to compare the human and plasmodium nucleosome stability, when assembled on a DNA fragment containing the 601 nucleosome positioning sequence [28]. Human histone octamers were reconstituted on the Cy5 labeled DNA fragment, whereas the plasmodium octamers were separately reconstituted on the Cy3 labelled DNA fragment (Fig 2B). Plasmodium nucleosomes like human nucleosomes recognize and are specifically positioned on the artificial 601 nucleosome positioning sequence. Both, parasite and human nucleosomes, form a defined nucleoprotein particle covering a single position on the 208bp long DNA fragment (S2A Fig).

The Cy3 and Cy5 labelled substrates were mixed to allow an internally controlled experiment, addressing nucleosome mobility with increasing temperatures (Fig 2B). Changes in nucleosome positions were analyzed on native polyacrylamide gels, scanning the individual fluorescence channels, followed by ethidium bromide staining to visualize the mixture of human and plasmodium nucleosomes. In contrast to the molecular dynamics simulation, the parasite octamers exhibited increased nucleosome sliding activity at elevated temperatures, when compared to the human nucleosomes. The result is suggesting weaker histone-DNA interactions within the plasmodium nucleosomes. As the 601 DNA presents an artificial, GC-rich sequence (56,9% GC), and the histones may be evolved to bind AT-rich DNA, we repeated the experiment with the AT-rich KahrP sequence amplified from the *P*. *falciparum* genome (14,6%; S2B Fig). Nucleosomes are formed on several locations on the DNA fragment resulting in a more fuzzy EMSA pattern and the pattern is distinct from human nucleosomes (addressed below). However, with this nucleosomal template thermal mobilization could be observed as well. Again, the plasmodium histones showed an increased thermal mobility, starting at lower temperatures (S2B Fig), suggesting a reduced stability of histone–DNA interactions.

Nucleosomes were shifted to slower migrating bands in the mobility shift assay, suggesting a relocation of the histone octamers to more central positions [29]. Still, to rule out that changes in band-shift position are not due to histone loss, we isolated nucleosomal bands from the gel and analyzed them for the equimolar histone ratio. Fuzzy nucleosomes and additional bands of the 601 nucleosomes contain the complement of all four histones (S2C Fig and Fig 5D and 5E), suggesting different nucleosome positions, rather than histone loss [25].

In order to test the stability of histone-DNA interactions we incubated the reconstituted nucleosomes with increasing concentrations of chloroquine and monitored the release of free DNA. Chloroquine is a DNA intercalating drug and its binding results in changing the geometry of DNA and stiffening the molecule. DNA bound proteins compete with chloroquine for binding, depending on their interaction strength. Proteins with relatively lower DNA binding affinity are displaced at correspondingly lower chloroquine concentrations [30]. Human and plasmodium nucleosomes were incubated with increasing concentrations of chloroquine, revealing the appearance of free DNA and the disruption of the plasmodium nucleosomes at 1mM chloroquine, whereas at least three times higher concentrations are required to initiate the disruption of the human nucleosomes (Fig 2C).

To analyze whether all histones or only a subset of the 4 histones reveal reduced histone-DNA interaction stability, we incubated nucleosomal arrays with increasing salt concentrations. The histones H2A and H2B can be dissociated from the particle starting at concentrations of 0.7M NaCl and above 1.2M NaCl also H3 and H4 start to dissociate from DNA [31,32]. Nucleosomal arrays were reconstituted on biotinylated DNA, bound to magnetic beads, incubated with increasing salt concentrations and the supernatants were collected (Fig 2D). Salt eluted proteins were analyzed by SDS-PAGE and visualized by silver staining. Correlating with the high number of amino acid changes in H2A and H2B, these two proteins exhibit significantly reduced binding affinities towards the DNA and the H3-H4 tetramer. At salt concentrations of about 0.7M NaCl, both, plasmodium H2A and H2B, were quantitatively eluted from the nucleoprotein particle, whereas their human counterparts still remained associated. In contrast, the plasmodium H3 and H4 proteins are stably bound to DNA, like the human H3/H4 proteins, even at high NaCl concentrations. Taken together the results show an overall decreased nucleosome stability, as compared to the human nucleosomes, due to the weaker interactions of H2A and H2B, resulting in the increased mobility of the nucleosomes on DNA.

### The evolution of parasite histones did not improve their binding to AT-rich DNA

The plasmodium genome has an average of 80.6% AT content and poly dA:dT sequences form straight and rigid helical structures with the potential to exclude nucleosomes [33–35]. It is shown that nucleosomes tend to form over GC-rich DNA with specific dinucleotide phasing [36–38].

**Fig 3:**
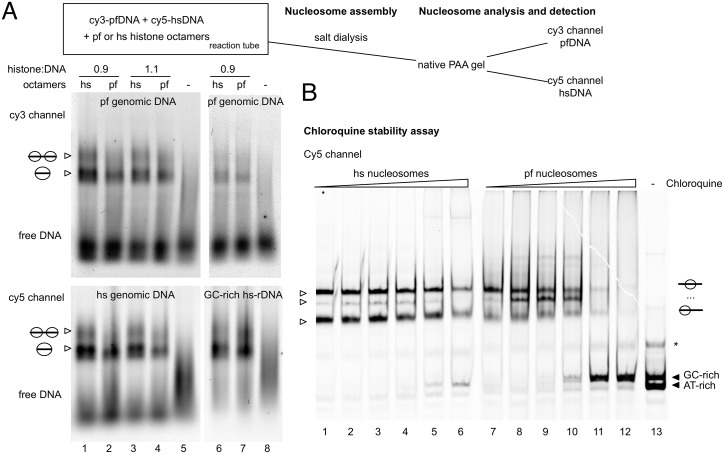
Analyzing the sequence dependent binding preferences of plasmodium and human histone octamers. (*A*) *P*. *falciparum* genomic DNA (19,6% GC-content), human genomic DNA (40% GC-content) and the GC-rich human rDNA sequence (60% GC-content) were isolated and fragmented by sonication. The purified DNA was chemically labelled with Cy3 (*P*. *falciparum* DNA) or Cy5 (human genomic DNA and human rDNA). Plasmodium DNA was mixed at equimolar ratios with the human DNA and used for chromatin assembly by salt dialysis, with either plasmodium (lanes 2, 4, 7) or human histone octamers (lanes 1, 3, 6) at different histone to DNA ratios (0.9:1 and 1.1:1). Chromatin assemblies were analyzed by native polyacrylamide gels and fluorescence scanning. The upper panel shows the cy3 channel, revealing the assembly of the plasmodium DNA, the lower panel depicts the results of the human genomic DNA and the human rDNA as indicated (cy5 channel). The efficiency of nucleosome assembly on AT-rich and AT-poor DNA can be judged by the ratios of free and reconstituted DNA in the individual lanes of the competitive assembly reactions. The free and nucleosomal DNA is indicated. (*B*) Chloroquine stability assay. The 210 bp KahrP DNA (AT-rich, Cy3-labelled, S3B Fig), the Cy5 labeled 601 DNA (GC-rich, 160bp) and a Cy5 labelled KahrP DNA fragment (AT-rich, 150 bp) reconstituted either with human (hs nucleosomes, lanes 1–6) or plasmodium octamers (pf nucleosomes, lanes 7–12) into nucleosomes and then mixed at equimolar ratios. Nucleosomes were incubated with increasing concentrations of chloroquine (0 to 9 mM), incubated for 10 min at room temperature and then analyzed by native polyacrylamide gel electrophoresis. The free DNA (lane 13) and the nucleoprotein complexes were visualized by fluorescence scanning as indicated. The asterisk shows the position of contaminating single stranded DNA.

To test whether plasmodium histones adapted to preferentially bind to AT-rich DNA, we fragmented plasmodium and human genomic DNA by sonification to create DNA fragments with a mean length of 200–300bp for nucleosome reconstitution experiments. The individual DNA samples were chemically labelled with fluorescent dyes, Cy5 for the human DNA and Cy3 for the plasmodium genomic DNA. The human and plasmodium genomic DNAs were mixed at equimolar ratios and reconstituted into nucleosomes by salt dialysis, using either plasmodium or human histone octamers (Fig 3A, lanes 1–5). The competitive assembly reaction revealed that the human GC-rich DNA was reconstituted more efficiently, by both, the human and plasmodium histones. The assembly efficiency was similar for human and plasmodium histones, showing no preference of the parasite histones for the AT-rich genomic DNA. In order to directly compare AT-rich plasmodium DNA with selected GC-rich DNA, we used the DNA sequence of the human ribosomal gene with a mean GC content of 60% (lanes 6 to 8). Again, both, the plasmodium and human histones bound the GC-rich DNA with higher affinity, fully assembling the GC rich DNA and only partially binding to plasmodium genomic DNA. Finally, we tested whether the high GC-content is required throughout the nucleosomal DNA for efficient nucleosome assembly. Three different PCR fragments, containing 147 bp, AT-rich (85,6%) DNA, flanked by DNA linkers with GC-content and increasing length, were created (S3A Fig). The parasite and human histone octamers again behave similar in that all three DNA molecules are reconstituted into nucleosomes with similar affinity. The result suggests that GC-rich DNA sequences at the border of the nucleosomes, where the H2A and H2B proteins contact DNA, are not sufficient to increase the GC-dependent binding affinity.

Next, we had a closer look at the available high throughput sequencing data and re-analyzed the datasets studying the accessibility of chromatin (Data sets used: SRX013309 [14], SRX013302 [14], SRX885811-SRX885819 [16]). Ponts and colleagues performed FAIRE assays [39] to reveal the accessible genomic regions, relative to their MNase resistant nucleosomal fraction. A genome browser snapshot of the FAIRE data, nucleosome occupancy and the GC content is given in S4A Fig. The accessible chromatin regions (FAIRE: median GC-content 15%; 19377 nucleosome free regions, blue line) and the sequences occupied by nucleosomes were quantified (nucleosomal: median GC-content 29%, 22770 nucleosome positions identified; red line). As control we used the same number of randomly chosen genomic regions (median GC-content 19%; grey line; S4B Fig). The data shows that intergenic regions are highly accessible, giving rise to large FAIRE domains, whereas the nucleosomes occupy the genic regions. The data suggests that nucleosomes are preferentially formed, or remain stable on GC-rich sequences that are mainly located in exonic regions. In contrast, nucleosomes are preferentially depleted from the AT-rich intronic and intergenic regions (S4C Fig). The FAIRE and MNase assays are complementary, suggesting more accessible chromatin at intergenic regions. However, these results are based on highly over-digested chromatin and it is known that MNase exhibits a strong preference for AT-rich sequences [40]. A recent genome wide study shows that nucleosomes in the intergenic regions are not depleted, but potentially disappear by over-digestion of chromatin with MNase [16]. Analyzing the new, low digested dataset, still revealed higher nucleosome occupancy at GC-rich sequences (S4D and S4E Fig). The enrichment of nucleosomes may reflect and correlate with the higher binding affinity of the nucleosomes towards GC-rich sequences (Data sets used: SRX885814 [16]), being an inherent feature of the parasite histones. Apparently, the *Plasmodium falciparum* histones did not evolve to efficiently package the AT-rich genome.

Using the chloroquine intercalation assay we now directly tested the stability of plasmodium and human nucleosomes on AT- and GC-rich DNA. Human and plasmodium nucleosomes were reconstituted on three different DNAs of varying AT-content, length and distinct fluorescent labels to monitor their chloroquine stability in a single reaction to allow direct comparison (Fig 3B; S3B Fig). The DNA molecules (a mixture of AT- and GC-rich DNA in Fig 3B; a 210 bp long AT-rich DNA in S3B Fig) were individually reconstituted into nucleosomes and then mixed, in order to perform an internally controlled competition assay to monitor the appearance of free DNA by chloroquine mediated nucleosome disruption. Interestingly human and plasmodium nucleosomes are similarly stable on the AT-rich DNA, giving rise to small amounts of disrupted nucleosomes with increasing chloroquine concentrations. However, on the GC-rich DNA different results were obtained, as revealed by the appearance of the free GC-rich DNA at lower chloroquine concentrations, as compared to the AT-rich DNA (Fig 3B). Plasmodium histones are less stably bound to GC-rich DNA than human nucleosomes, suggesting that changes in the plasmodium histone sequence result in nucleosome de-stabilization.

### Decreased nucleosome stability does not correlate with an increase in ATP dependent nucleosome dynamics

**Fig 4:**
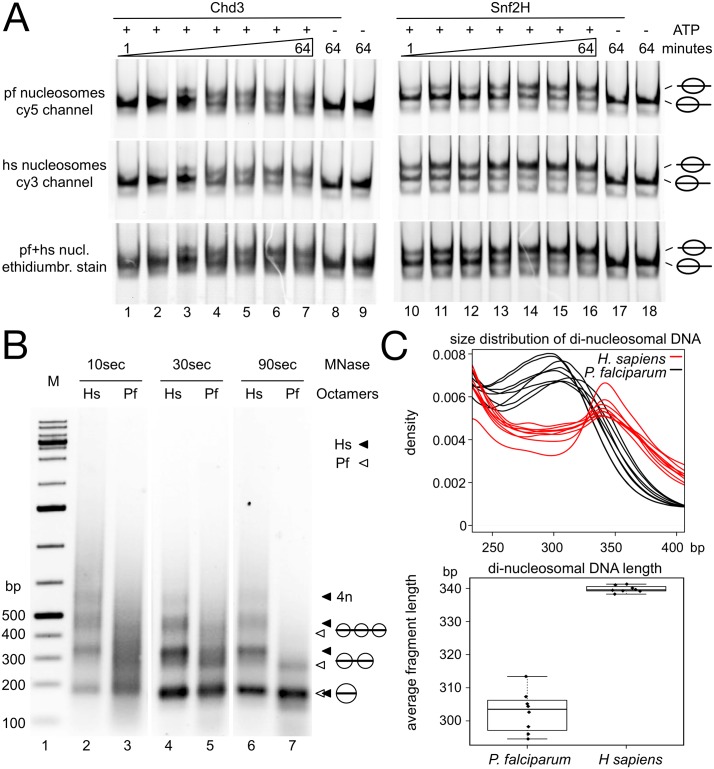
Analysis of nucleosome remodeling and nucleosome spacing. (*A*) Competitive nucleosome remodeling reactions. In the same reaction Cy5-labelled plasmodium nucleosomes (upper panel) and Cy3-labelled human nucleosomes (middle panel) reconstituted on 601 DNA, positioned at the border of the 208bp long DNA fragment, were incubated with increasing concentrations of recombinant Chd3 (lanes 1 to 9) or Snf2H (lanes 10 to 18). Reactions were incubated for 60 min at 37°C in the presence or absence of ATP, as indicated. Nucleosome positions were analyzed by EMSA and imaged for the Cy5 and Cy3 channel, respectively. The lower panel shows the total nucleosomal reaction after ethidium bromide staining. The positions of the nucleosomes are indicated on the right side. (*B*) Recombinant histone octamers of the indicated type (human = Hs, plasmodium = Pf) were used for chromatin assembly with a 11kb plasmid by salt dialysis. Chromatin was subjected to partial MNase digestion (10 to 90 sec), stopping the reaction by the addition of SDS/EDTA. The DNA fragments were purified and visualized by agarose gel electrophoresis and ethidium bromide staining. The positions of the mono-, di- and tri-nucleosomal DNAs are indicated by black triangles marking the human and white triangles marking the plasmodium complexes. (*C*) Di-nucleosome density and box plots revealing the nucleosome repeat lengths *in vivo*. The size distribution of di-nucleosomal fragments derived from human (red) as well as plasmodium DNA (black) after MNase digestions (SRX885811-SRX885819)[16]. MNase digestions were performed at different stages of the erythrocytic life cycle of *P*. *falciparum*. Each stage of the life cycle is plotted individually together with the corresponding human DNA fraction.

Cellular nucleosomes are mobilized by ATP dependent chromatin remodeling enzymes that use the energy of ATP hydrolysis to move the histone octamer on the underlying DNA [41,42]. Next, we tested whether the increased mobility and reduced stability of plasmodium nucleosomes would also affect their dynamics in an enzyme catalyzed remodeling reaction. Nucleosome remodeling reactions were performed with recombinant remodeling enzymes from the Chd and ISWI families; i.e. Chd3 and Snf2H (Fig 4A). Again, Cy3 and Cy5 labeled 601 DNA, either reconstituted with the plasmodium or the human nucleosomes were mixed in one test-tube to allow the direct comparison of the remodeling kinetics. Chd3 (Fig 4A, lanes 1 to 9) exhibited very similar remodeling kinetics on both types of substrate. The nucleosomes positioned at the border of the DNA fragment were partially moved to the center of the DNA fragment with no apparent difference of positioning and remodeling efficiency. To our surprise, the human ISWI type remodeler Snf2H did move the parasite nucleosomes less efficient than the human nucleosomes (Fig 4A, lanes 10 to 18). The less stable plasmodium nucleosome is a worse substrate for Snf2H, suggesting that Snf2H either binds the nucleosome with lower affinity, or nucleoprotein structures are lacking that are required for Snf2H-dependent remodeling.

We first tested the binding affinity of Snf2H towards the Cy5-plasmodium and Cy3-human nucleosomes in competitive bandshift analysis (S5A Fig), where we did not observe differences. Next, we had a closer look at the histone H4 tail. ISWI type remodeling enzymes strictly depend on the intact H4 tail and more detailed on the amino acids R17-H18-R19 of the H4 tail [43,44]. A closer inspection of the plasmodium histone H4 revealed the presence of the RHR motif. However, at position 21 plasmodium H4 exhibits a V to I change in sequence that could influence remodeling efficiency (S5B Fig). In order to test if this is the case, we prepared hybrid octamers (H4hyb) containing the human H4 in combination with the plasmodium histones H2A, H2B and H3 (Fig 1A, lane 8). Hybrid nucleosomes were compared in the competitive remodeling assays with the human nucleosomes, showing that the amino acid exchange at position 21 affects the recognition of the histone H4 tail (S5C Fig). Remodeling is similar efficient when comparing the hybrid nucleosomes with the human counterpart, suggesting that the reduced stability of the parasite nucleosomes does not automatically increase the ATP dependent nucleosome remodeling rate.

### Plasmodium histone octamers form tightly spaced nucleosomal arrays

Nucleosomes form arrays with defined linker lengths on DNA. The length of the DNA linker is cell type specific in higher eukaryotes and also reveals organism-specific inter-nucleosomal distances [45]. Partial MNase digestions of native *P*. *falciparum* chromatin exhibits a nucleosomal ladder, revealing the regular array of nucleosomes on DNA [11,46]. We tested whether the parasite histones would form similar nucleosomal arrays as the human histones, when reconstituted on circular, supercoiled DNA by the salt dialysis method (Fig 4B). Plasmodium histones form nucleosomal ladders, but interestingly, the plasmodium nucleosomal ladder exhibited repeatedly a more smeary appearance, but still revealing the repetitive pattern that corresponds to the array-form. The smeary appearance could be explained by a reduced nucleosomal stability, corresponding to increased MNase sensitivity of the nucleosome core, and/or an increased heterogeneity of DNA linker lengths. However, in contrast to the spacing of the human nucleosomal array, the plasmodium nucleosomes obey significantly closer inter-nucleosomal spacing, with a mean distance of about 155bp. To rule out effects of reduced nucleosomal stability, associated with the generation of DNA fragments of sub-nucleosomal size, we performed extended MNase digestions and detailed DNA fragment length analysis (S6A and S6B Fig). Prolonged MNase digestions gave rise to stable digestion intermediates of about 150bp in size (S6B Fig) and bioinformatic analysis, of the mono-nucleosomal DNA fragment size distribution in the Kensche data set, did also reveal a bona fide protection of about 150 bp of DNA by the histone octamers (S6C Fig). Even though our assays suggest a reduced stability of the plasmodium nucleosome, the histone octamer does stably protect the 147bp nucleosome core sequence from MNase digestion, as shown for the human nucleosome core.

Next, we used the experimental data of Kensche and colleagues to mine for di-nucleosomal DNA fragments that are abundant due to their low MNase digestion regimen. In order to have internal controls, we applied the bioinformatic analysis to all 8 erythrocytic stages and extracted the plasmodium and human DNA fragments from the data. The fragment sizes were plotted and di-nucleosomal fragment lengths were calculated (Fig 4C). Indeed, we find the same short inter-nucleosome distances *in vivo* as *in vitro*, and longer nucleosome repeat lengths (NRL) for the human nucleosomes. Our *in vitro* analysis and the *in vivo* data show that the NRL is even shorter than in yeast, the organism with the shortest repeat length known so far. Taken together our data suggests that the global NRL distribution in the genome is driven by the biochemical properties of the histones and not by additional cellular activities. Interestingly, experimental and modeling studies have shown that short NRLs (up to 177bp) inhibit the folding into higher order structures of chromatin, being in well agreement with the accessible chromatin of *P*. *falciparum* [47].

### Plasmodium histone octamers exhibit reduced sequence dependent nucleosome positioning capabilities

DNA sequence directs nucleosome positions *in vivo*, as the histone octamer does preferentially bind with a ~10bp periodicity to anti-phased A/T and G/C dinucleotides, corresponding to the helical turn of DNA wrapped around the histone core [9,36,48].

**Fig 5:**
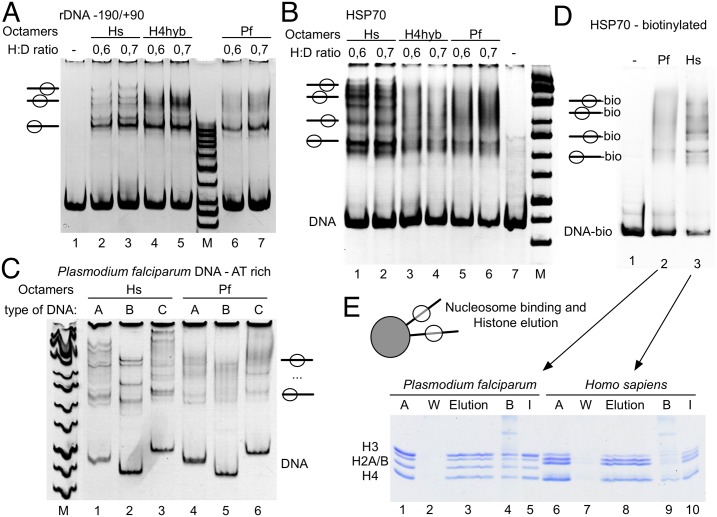
*P*. *falciparum* nucleosomes override nucleosome positioning signals. (*A*) Sequences encompassing the mouse rDNA promoter from position -190 to +90 were amplified by PCR and used as substrate for nucleosome assembly. Free DNA (lanes 1), nucleosome assembly reactions using the recombinant human histone octamers (lanes 2 and 3), octamers consisting of the plasmodium H2A, H2B, H3 and human H4 (H4hyb, lanes 4 and 5), the plasmodium octamers (lanes 6 and 7) and a size marker (M) were separated by native polyacrylamide gel electrophoresis and stained with ethidium bromide. Nucleosome positions and the respective histone to DNA ratios used for assembly are indicated. (*B*) Experimental setup like in (*A*) but a 370-bp fragment carrying the *D*. *melanogaster* HSP70 promoter was used for assembly. (*C*) Nucleosome assembly was performed on three different AT-rich sequences (A, B and C) amplified from the *P*. *falciparum* genome (Chromosome 11, position 1307500–1308399). The sequences were reconstituted into nucleosomes either with recombinant human or plasmodium histone octamers and analyzed by native polyacrylamide gel electrophoresis. (*D*) To analyze whether the full complement of histones assemble on DNA, the biotinylated HSP70-DNA (sequence used in *B*), was reconstituted into nucleosomes and separated on native polyacrylamide gels (lanes 2 and 3). (*E*) Plasmodium and human nucleosomes shown in (*D*) (I: Input—lanes 1 and 10) were coupled to magnetic beads and washed twice with 150 mM NaCl buffer (W: Wash—lanes 2 and 7). Histones were eluted with 2M salt (Elution–lanes 3 and 8), the individual fractions and proteins remaining bound to the magnetic beads (B: Beads–lanes 4 and 9) were analyzed by SDS-PAGE and Coomassie Blue staining.

As documented above, we used the 601 DNA sequence, representing an artificial high affinity binding site for nucleosomes, to prepare nucleosomes with defined positions (Fig 2B). However, we noticed that on natural DNA sequences no discrete positioning is obtained and that the reconstitution pattern deviates from the human nucleosomes. To unravel the underlying reason, we used a set of native mouse (rDNA promoter -190/+90), *D*. *melanogater* (HSP70) and genomic *P*. *falciparum* sequences (Pf3D7v3: 1307500–1308399; S7A Fig) to visualize and study nucleosome positioning. DNA fragments were reconstituted into nucleosomes, using either recombinant human or parasite histone octamers. As expected, the human histone octamers form discrete nucleosome positioning patterns on all DNA templates used, being visible as specific bands in the native polyacrylamide gels (Fig 5A–5C). Multiple bands arise from different, specific mono-nucleosomal positions on the given DNA molecules. As previously shown, the nucleosomal patterns do depend on the sequence and structure of the DNA molecule [49]. In contrast, plasmodium nucleosomes exhibit a smeary appearance, with only a few prominent bands in the electromobility shift assay. The results suggest that the majority of the octamers do not assemble on discrete, preferential sites, but are randomly distributed along the DNA. Discrete bands are most often the lowest bands, corresponding to a nucleosome covering the thermodynamically stable end position of the DNA [49]. The loss of nucleosome positioning is apparently driven by the changes in the amino acid sequences of the plasmodium histones, but independent of histone H4, as shown by using a plasmodium hybrid octamer, carrying the human histone H4 (Fig 5A and 5B).

The fuzziness of the assembly reaction is most probably not an effect of weaker histone DNA interactions and DNA breathing in the nucleosome, as MNase digestions show that the DNA entry/exit sites of the nucleosomes are stably protected from nuclease cleavage (S6A and S6B Fig). These experiments do also reveal the lack of detectable sub-nucleosomal, or non-canonical histone-DNA complexes. In addition, plasmodium histone octamers form intact nucleosomes on DNA, as we have shown by assembly on the 601 DNA, and these nucleosomes are properly structured and stoichiometric, as revealed by the enzymatic nucleosome remodeling assays (Fig 2B; S2C and S4A Figs). In order to show that this is also the case for the experiments shown here and to exclude nucleosomal dis-integration to be responsible for the lack of positioning, we analyzed the histone content of the reconstituted nucleosomes (Fig 5D and 5E). The biotinylated HSP70 DNA was reconstituted into nucleosomes (Fig 5D) and the bound histones were eluted from DNA, after binding it to magnetic beads. Even though there is no clear nucleosomal pattern, all core histones are present in stoichiometric amounts (Fig 5E), suggesting the formation of bona fide nucleosomes. This result was also confirmed by the salt elution experiment performed with the nucleosomal arrays (Fig 2D).

Our observation is supported by *in vivo* data, when analyzing the plasmodium nucleosome positions from the different genome wide studies. The high throughput sequencing dataset of Bunnik and colleagues contains contaminations of human nucleosomal DNA (3.2 mio reads) in addition to the plasmodium DNA sequences, serving us as internal controls for the bioinformatic analysis [15,50]. We selected nucleosomal DNA fragments, ranging in size from 146 to 148bp, and analyzed their nucleotide frequency along the DNA path (61,263 plasmodium and 121,819 human sequences). As shown in previous studies, the human nucleosome positions exhibit an enrichment in G/C every 10bp and shifted by 5bp an enrichment in A/T di-nucleotides [9,51] (S7B Fig), revealing sequence dependent nucleosome positioning signals. In contrast, this periodicity is lacking in the DNA sequences of the plasmodium nucleosomes, suggesting that the plasmodium octamers do not obey the same positioning rules as the human histones. Like in our *in vitro* positioning experiments, the *in vivo* analysis shows that the parasite histones do not recognize the sequence dependent nucleosome positioning signals. As this dataset was derived from heavily digested chromatin, we also re-analyzed the dataset of Kensche and colleagues with our bioinformatics pipeline [16]. These authors show that nucleosome positioning can be observed at regulatory regions, but is rarely detected in the intergenic regions [16], being consistent with our experimental data. For a detailed analysis, we isolated the contaminating human nucleosomal DNA to visualize the oscillation of GC and AT sequences every 10bp (S7C Fig). The sequence oscillation of human nucleosome occupancy can be observed, but due to the relatively low number of read counts the pattern is similar, but not identical to the Bunnik study. In contrast to the Bunnik data, the 146-148bp long DNA fragments of *P*. *falciparum* did reveal a weak oscillation of the AT-dinucleotides, with an unusual enrichment at the dyad axis and additional AT-peaks shifted by 5bp. However, no oscillation of the CG-dinucleotides could be observed. The pattern is not completely lost as suggested in the dataset of the LeRoch manuscript, but very different and relaxed in comparison to the human dinucleotide pattern. Differences in the dinucleotide repeat pattern between human and plasmodium nucleosomes can be also attributed to the high AT-content of the plasmodium genome, albeit clearly shifted peak distributions indicate intrinsic differences in motif recognition. In addition, the absence of positioned nucleosomes in the genome do confirm relaxed recognition of nucleosome positioning signals, like the side by side comparison of human and plasmodium nucleosome positioning patterns. The data suggests that plasmodium histones either recognize different sequence dependent signals, or have a reduced affinity to DNA motifs being located in the realm of the nucleosome. In order to reveal an alternative nucleosome positioning pattern in detail, future experiments would have to reconstitute plasmodium nucleosomes on whole genomic DNA libraries *in vitro* and perform sequence analysis of the MNase protected DNA fragments. Our biochemical studies do provide an explanation for the lack of positioning patterns *in vivo*, arguing for the presence of alternative and additional sequence-dependent positioning signals in the regulatory regions of *P*. *falciparum*.

### Poly dA:dT boundaries are required to position nucleosomes in the plasmodium genome

**Fig 6:**
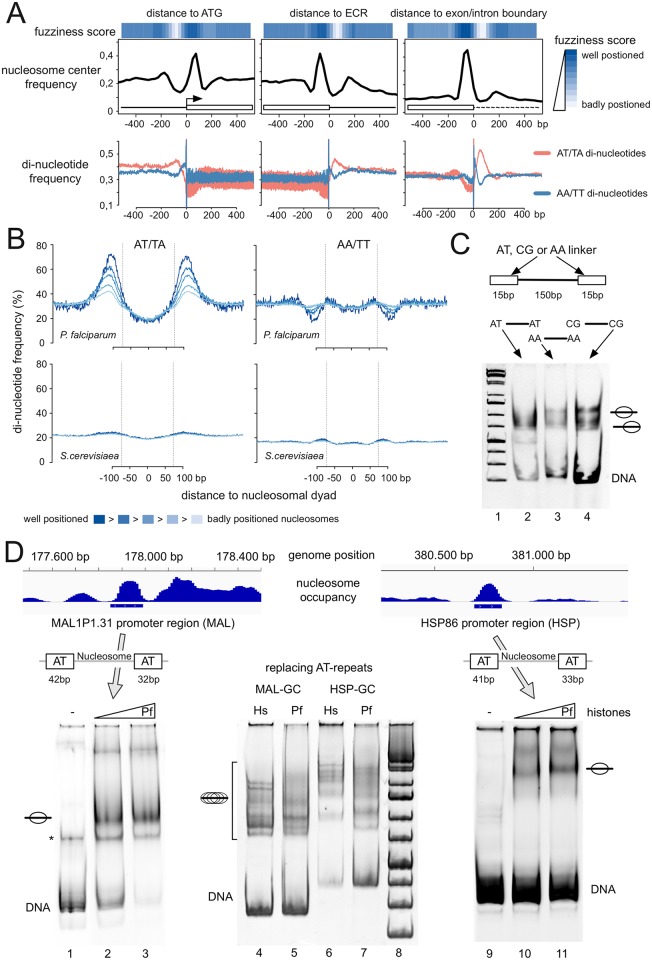
AT-repeat containing sequences in the linker region of the nucleosome determine the position of plasmodium histone octamers. (*A*) Nucleosome positioning at ATG (beginning of the protein coding region), ECR (end of protein coding region) and exon/intron boundaries of all annotated *P*. *falciparum* genes were aligned. Individual nucleosome positions were further characterized by a fuzziness score and the average fuzziness signal for all aligned genes was plotted as a heatmap (top). The color code is indicated on the right. The average frequency of identified nucleosome midpoints (middle) and of AT/TA (red) and AA/TT di-nucleotides (blue) over regulatory regions of all annotated genes is given. (*B*) Changes in AT/TA and AA/TT di-nucleotide frequencies within the nucleosome and its linker regions compared to the fuzziness score of nucleosome positioning. Five different fractions of nucleosomes, from well positioned (dark blue) to badly positioned (light blue) nucleosomes for *P*. *falciparum* (top panel) and *S*. *cerevisiae* (bottom panel) were analyzed. Vertical lines indicate nucleosome boundaries. The color code for the fuzziness score is indicated below. (*C*) A 150bp long HSP70 sequence was flanked by either two 15bp long AT-rich (AT), AA-homopolymers (AA) or GC-rich (CG) sequence linkers and amplified by PCR. The indicated PCR fragments were used for nucleosome assembly with plasmodium histones and analyzed on 5% native polyacrylamide gels. The templates used for assembly and the nucleosome positions are indicated. (*D*) Two genomic regions exhibiting positioned nucleosomes containing native AT-repeat flanking sequences (MAL and HSP) were cloned, amplified by PCR and used to analyze nucleosome positioning *in vitro*. MAL (lanes 1–3), HSP (lanes 9–11) and the MAL and HSP PCR fragments having the AT repeats replaced by GC-rich sequences (MAL-GC; HSP-GC; lanes 4–7) were used for nucleosome assembly with plasmodium (Pf) and human histones (Hs). Nucleosome positioning was analyzed on 5% native polyacrylamide gels. The free DNA, contaminating single stranded DNA (marked by an asterisk), positioned and delocalized nucleosomes are shown.

As the parasite nucleosome lost its capability to adopt sequence-specific nucleosome positioning, we analyzed which signals could determine the positioning of nucleosomes at regulatory regions. We tested whether the associated linker DNA sequences are involved in the translational positioning of nucleosomes. Using the genome wide nucleosome occupancy data, we defined a fuzziness parameter of nucleosome positioning. The idea is that well positioned nucleosomes create a small occupancy footprint on genomic DNA, with narrow peaks of nucleosome read annotations. In order to calculate the degree of fuzziness we used the established DANPOS2 toolkit [52]. Next we aligned the *P*. *falciparum* genes according to the start of the protein coding region (ATG), the end of the coding region (ECR) and the exon/intron boundaries, plotting nucleosome occupancy and the degree of nucleosome fuzziness above. We have performed the analysis for the datasets available from the LeRoch and Bártfai labs, as described above, giving similar results (Fig 6A and 6B and S8A and S8B Fig respectively).

Even though the nucleosome core exhibits relaxed recognition of sequence dependent nucleosome positioning signals, we observed specifically positioned nucleosomes at these regulatory regions. An inspection of the AA/TT and AT/TA dinucleotide frequency revealed their non-random distribution around the strongly positioned nucleosomes. AT dinucleotide frequencies are enriched, whereas AA dinucleotide frequencies are reduced in the linker regions of these nucleosomes (Fig 6A; S8A Fig). Next we sorted the genomic peaks of nucleosome occupancy according to the fuzziness parameter and plotted the respective AA and AT dinucleotide abundance (Fig 6B, S8B Fig). As shown for the site-specific analysis, the best positioned nucleosomes are characterized by the largest depletion of AA/TT di-nucleotides and the strongest enrichment of AT/TA di-nucleotides, flanking the nucleosome core. The bioinformatics analysis clearly reveals that flanking sequences of well positioned parasite nucleosomes exhibit increased stretches of AT-rich elements, suggesting the importance of linker DNA in translational nucleosome positioning. In contrast, yeast nucleosomes do not reveal such linker DNA dependent positioning signals (Fig 6B).

In order to test this behavior experimentally, we generated three DNA fragments with a 150bp long HSP70 sequence, flanked either by DNA linkers consisting of 15 bp of AT di-nucleotides, AA di-nucleotides or GC-rich sequences (Fig 6C). The three different DNA fragments were used for nucleosome assembly and analyzed on native polyacrylamide gels. Whereas the AA- and GC-rich linkers favor the assembly of nucleosomes at more than two distinct sites, the AT linker containing DNA fragment reveals a distinct positioning pattern with a reduced number of positions. Still the 15bp of flanking DNA did not convincingly determine a unique translational position, showing that the 15bp long AT repeat is not a sufficiently strong signal. However, *in vivo* the best positioned nucleosomes are flanked by AT-repeats of up to 50 bp, suggesting that longer sequence elements are required.

Next we used the genomic data to retrieve highly positioned nucleosomes in plasmodium. We selected sequences close to the MAL1P1.31 (MAL) and HSP86 (HSP) promoter, harboring a positioned nucleosome. The genomic regions were cloned and amplified by PCR, containing AT-rich linker regions from 32 to 42bp. DNA fragments were used for nucleosome assembly (Fig 6D). Indeed, these DNA fragments revealed only one main nucleosomal position in the EMSA assay, showing that long AT-repeats are required to guide nucleosome positioning *in vitro* and potentially *in vivo* (lanes 1–3 and 9–11). To prove that the AT-sequences are responsible for positioning within these genomic sequence contexts, we replaced the AT-repeats by GC-rich DNA elements. The different DNA templates were reconstituted in parallel and revealed that in the absence of the AT-repeats discrete nucleosome positioning is lost (lanes 4–7). Our results suggest that plasmodium histone octamers have a reduced affinity for AT di-nucleotide repeats containing DNA elements and are therefore forced to assemble next to these sequences. In summary, we show that the biochemical properties of the histone octamers dictate the positioning of the parasite nucleosome cores and the accessibility of the chromatin structure.

## Discussion

The sequences of plasmodial histones are highly divergent from those of other eukaryotes. We questioned whether this difference represents an adaptation to the extraordinarily high AT-content of *P*. *falciparum* and whether these amino acid replacements do alter the physicochemical properties of the nucleosome. The results presented in this study are un-expected, showing that the observed mutations do not result in better binding of AT-rich DNA. In contrast, we even observe a reduced stability of the nucleosomes on GC-rich DNA, accompanied by a reduction in the thermal stability of the octamer on DNA. In agreement with the reduced thermal stability, we also observe reduced salt stability of H2A and H2B, the histones with the majority of sequence alterations.

The plasmodium nucleosomes lost their strong ability to recognize the phased AT and GC di-nucleotide patterns, weakening the intrinsic capability of sequence dependent nucleosome positioning. The *in vitro* reconstitution experiments are backed up by the comparative genomic analyses of the human and plasmodium nucleosome positions *in vivo*, showing either no (LeRoch data), or a strongly reduced and altered recognition of di-nucleotide patterns (Bártfai data). Still, positioned nucleosomes can be detected at regulatory regions like the transcription start sites, transcription termination sites and intron/exon boundaries. We can show that nucleosome positioning at these sites is achieved by the presence of long AT-repeats in the linker regions of these nucleosomes, being unfavorable sites and therefore placing the nucleosomes next to such sequences. These AT-rich sequence boundaries are preferentially located at regulatory elements in plasmodium, but not in yeast and human genomic DNA, presenting an alternative mechanism of nucleosome positioning.

Eukaryotic nucleosome cores are spaced by 20 (budding yeast) to 75bp (echinoderm sperm) of linker DNA connecting neighboring nucleosomes [45]. The plasmodial nucleosomal arrays reconstituted in this study reveal the shortest linker lengths in the eukaryotic kingdom known today. With a repeat length of about 155bp, nucleosome spacing is significantly shorter than in yeast. Published *in vivo* data is conflicting, with a study suggesting repeat lengths of 180bp [53] and others that correlate well with our results, describing extremely short nucleosome repeat lengths of 155bp (+/-5bp) [11,46]. In our opinion, the combined biochemical and genomic data analysis does convincingly reveal the short NRL that is present throughout the erythrocytic stages of the plasmodium life cycle. We suggest that sequence alterations in the histones allow the generation of compact nucleosomal arrays determining the unique chromatin architecture of *P*. *falciparum*. Like yeast, *P*. *falciparum* is lacking histone H1 and our experiments show that the short NRLs are a result of the biochemical properties of the plasmodial histone-octamer rather than depending on the absence of H1 binding. Like the yeast genome, the plasmodial genome is relatively accessible, as judged by MNase and FAIRE assays [14] implying the lack of condensed heterochromatin structures. Several reports and modeling studies show that short nucleosome spacing interferes with the formation of organized higher order structures of chromatin, resulting in an accessible genome architecture [54,55]. Our data suggest that plasmodial histones evolved to enable short nucleosome repeat lengths potentially inhibiting the formation of compact higher order structures of chromatin.

Besides the atypical spacing, plasmodial histones exhibit reduced thermal and salt stabilities revealing weakened histone DNA interactions. The abridged levels of higher order structures of chromatin is accompanied with reduced nucleosome stability, potentially simplifying the access of sequence specific binding proteins to DNA. Kensche and colleagues showed that during the transition between the cell cycle stages chromatin structure and nucleosome positioning changes occur around the transcription factor binding sites [16].

Screens of histone mutations in yeast, relieving the dependence of transcription on the SWI/SNF remodeling complex (SIN) identified specific changes in the histones H3 and H4 [56,57]. The amino acid changes are preferentially located at SHL locations, where the histones do directly interact with DNA and do alter nucleosome stability and chromatin compaction [58,59]. The *P*. *falciparum* H3 and H4 sequences do not exhibit these classical SIN mutations. But, the H2A and H2B histone sequences exhibit clusters of amino acid changes at and close to SHL locations, with two sites being equivalent to SIN mutations (G to T in H2B position 72 and T to S in H2A position 76). The role of H2A and H2B SHL regions in the stabilization of the nucleoprotein structure was not yet analyzed, but they are suggested to play an important role [60]. Interestingly, the *P*. *falciparum* histones exhibit clustered amino acid changes at the SHL3.5 and 4.5 regions of H2B and H2A that could alter nucleosome stability and contribute to the open chromatin structure in *P*. *falciparum*.

According to our *in silico* assessment of protein-DNA interactions, the interactions of human and plasmodial histones with DNA are indistinguishable, which is in contrast to our experimental results. How can one reconcile these seemingly conflicting findings? Due to their flexibility, N- and C-termini of the histones were not considered in homology modeling; consequently, our binding analysis was blind for their putative interactions with DNA and effect on nucleosome stabilization. On the other hand, a comparison of human and plasmodial histone sequences reveals drastic differences in these histone termini (Fig 1 and S1 Fig). We suggest a crucial contribution of these flexible histone termini to DNA binding and nucleosome stabilization, which is a hypothesis to be tested in future experiments.

The mechanism driving nucleosome positioning is an essential field of study in chromatin research, as the locations of the nucleosomes on DNA determine the accessibility for sequence specific DNA binding proteins. The first description of nucleosome positioning *in vivo* and revealing the important role of multiple histone-DNA interactions and DNA structure in mediating positioning [61,62] initiated a search for DNA dependent positioning signals. Many structure based differences in DNA sequence patterns, like AA [62], GG [63,64], TA and GC oscillations, several tri- to poly-nucleotide patterns (for a review see ref. [65]), motifs being essential for anisotropic DNA bending (for a review see ref. [66]) were proposed to determine the intrinsic nucleosome positioning behavior. In principle, sequences that are already pre-curved, requiring low energy levels to wind around a histone octamer should preferentially bind and position nucleosomes. Models were devised to predict nucleosome positioning *in vitro* and *in vivo* from DNA sequence [9], albeit the predictive power is currently being questioned [67].

With the study presented here, we further question the sequence dependent view of nucleosome positioning. The overall structure of the nucleosome of *P*. *falciparum* nucleosomes is identical to other eukaryotic structures, but has a strongly reduced ability to recognize sequence encoded nucleosome positioning signals *in vivo* and *in vitro*. Phased di-nucleotide repeat patterns, clearly detectable for the human nucleosomes, are mostly diminished in plasmodium. Biochemical reconstitution of nucleosomes on DNA templates of different origin and AT content only exhibit a few discrete nucleosome positions. The lack of positioning argues that additional constraints must exist to determine nucleosome positioning. As the exchange of human histone H4 for the plasmodial H4 in the octamer did not restore positioning, the signals must be read by the other histones. The best candidates are the histones H2A and H2B that exhibit the most differences to the other canonical histones (Fig 1). We hypothesize that the histone tails, bearing most of the amino acid exchanges, may interact with DNA and influence nucleosome positioning, but details have to be addressed in future experiments.

Lack of intrinsic nucleosome positioning capabilities are exchanged by linker DNA dependent nucleosome positioning mechanisms in *P*. *falciparum*, like the AT repeats enriched in the associated linker DNA of well positioned plasmodial, but not in yeast and human nucleosomes. A closer look also reveals that such external positioning sequences do flank regulatory regions and thereby ensure specific positioning of regulatory nucleosomes.

## Materials and Methods

### DNA and proteins

Plasmids encoding the canonical human and plasmodium histone sequences were optimized for bacterial expression and ordered as synthetic genes. Recombinant expression, purification of histones from inclusion bodies and octamer refolding was done as described previously [68]. Recombinant SNF2H, Chd1, Chd3 and Chd4 were expressed in Sf21 cells (Invitrogen) and prepared according to standard procedures [49].

DNA fragments were synthesized by PCR, using fluorescently labelled oligonucleotides binding to the described murine rRNA gene promoter (-190 to +90, relative to the transcription start site), to the *D*. *melanogaster* HSP70 promoter and to the DNA 601 sequence. The AT rich *P*. *falciparum* sequence Pf3D7v3:1307900–8200 was subcloned and DNA fragments were prepared by PCR or restriction enzyme digestion and purification. MAL and HSP sequences were generated by oligonucleotide-annealing, -ligation and cloning into pUC19. A plasmid containing the sequence encompassing the Knob-associated histidine-rich protein (KahrP) gene promoter, was kindly provided by Till Voss.

### Nucleosome assembly

Nucleosomes were assembled according to Rhodes and Laskey using the salt gradient dialysis technique [69]. A typical assembly reaction (50 μl) contained 4.0 μg DNA, varying amounts of recombinant histone octamer, 200 ng BSA/ml, in high salt buffer (10 mM Tris, pH 7.6, 2 M NaCl, 1 mM EDTA, 0.05% NP-40, 2 mM ß-mercaptoethanol). The salt was continuously reduced for 16–20 h and nucleosomes were assayed in 80 mM salt buffers. The quality of the assembly reaction was assayed by electromobility shift assays on native polyacrylamide gels or by partial MNase digestion and analysis of the nucleosomal ladder on agarose gels.

### Thermal mobilization, salt elution of nucleosomes and assaying nucleosome stability

Mobility shift assays utilizing thermally induced movement of nucleosomes were carried out as described [70]. Nucleosomal DNA, either labelled with Cy3 or Cy5 (150ng of each template) were incubated in a total volume of 20 μl Ex80/BSA-buffer (10 mM Tris, pH 7.6, 80 mM NaCl, 1.5 mM MgCl_2_, 1 mM EDTA, 0.05% NP-40; 200 mg/l BSA) for 60 min at 48° to 66°C. Nucleosome positions were analyzed on a native 6% polyacrylamide gels (0.4x TBE) and visualized fluorescence scanning.

To monitor nucleosomal stability with increasing chloroquine (Sigma) concentrations, differentially fluorescent AT-rich and GC-rich DNA fragments were fully reconstituted into nucleosomes. Nucleosomal species were mixed to allow competitive and comparative assays. Increasing concentrations of chloroquine were added to the nucleosomes in EX80/BSA-buffer and incubated for 10 min at 37°C. Nucleosome positions were analyzed as described above.

Biotonylated DNA was prepared by PCR, using one biotinylated primer and the plasmid pUC19 as a template. Plasmodium and human nucleosomal arrays were reconstituted on the 2375bp long DNA fragment by the salt dialysis method. Nucleosomal arrays (4μg) were coupled to magnetic streptavidin coated Dynal Beads and unbound DNA was washed with Ex80-buffer. Chromatin was incubated with LO-buffer, stepwise increasing the NaCl concentration from 400 to 1400 mM, then bound to the magnetic beads and the supernatant was collected. Salt eluted histones were analyzed by SDS-PAGE (17%) and visualized by silver staining.

### Nucleosome remodeling assay and electromobility shift assays

Nucleosome mobility was assayed as described [49]. Briefly, reactions contained 20 nM Cy5 and Cy3 labelled DNA reconstituted into nucleosomes, 1 mM ATP, 100 ng/μl BSA, in Ex80 buffer (20 mM Tris pH 7.6, 80 mM KCl, 1.5 mM MgCl_2_, 0.5 mM EGTA) and recombinant remodeling enzymes. Nucleosomes were incubated with the enzymes for 60 min at 30°C. The reactions were stopped by the addition of 1μg of plasmid DNA and incubated for 5 min on ice. The nucleosome positions were analyzed by electrophoresis on 6% native polyacrylamide gels in 0.4x TBE and fluorescence scanning. Nucleosome remodeler interactions were probed by the incubation of 20nM of nucleosomes with increasing concentrations of remodeling enzymes, for 60 min at 30°C, then loaded on 6% native polyacrylamide gels in 0.4x TBE and fluorescence scanning.

### Sequence mapping

Sequence data from formaldehyde-assisted isolation of regulatory elements (FAIRE; SRX013302) and MNase mediated purification of mononuclesomes (SRX013309, SRX316306, SRX316307, SRX885811-SRX885819) [14–16] was downloaded and mapped to the *P*. *falciparum* 3D7D genome version 3, or the UCSC human genome version 37 (hg19) using the local alignment option of bowtie2 with default settings [71]. The reads were filtered for mapping quality (MAPQ > 20) and concordant alignment in the case of using paired-end sequencing data. The bowtie output was further processed using SAMtools [72] and BEDtools [73] for customized analysis purposes.

### Di-nucleotide frequency in the nucleosome core

Human as well as plasmodium derived nucleosomal fragments with a size of 146-148bp were selected (SRX316307, SRX885814) [15,16]. Nucleotide frequency was calculated using annotatePeaks.pl script from the HOMER software package [74] with the following parameters: -hist 1 –di–size -150,150.

### GC content analysis

The findPeaks script from the HOMER software package was applied to FAIRE (SRX013302) [15] and MNase single-end sequencing data (SRX013309) with the following parameters: -style histone–size 147 –region–norm 1e7 –gsize 23292104 –minDist 20 –inputSize 147 –C 0 –F 2 –minTagThreshold 50. To determine the nucleosome peaks the MNase sequencing data was used as input and the FAIRE sequencing data as background (vice versa for the FAIRE peaks). Peaks were centered at the position with the highest read coverage using getPeakTags script (-start 80 –end 80 –center–fragLength 70). Peaks overlapping more than 100bp were merged and GC content of peaks was calculated using BEDtools. annotatePeaks.pl script was applied to annotate the peaks to genomic features (exon, intron, intergenic) on the plasmodium genome.

### Computational analysis of nucleosome positioning

Nucleosome positions of MNase paired-end sequencing data (SRX316306, SRX885814) [15,16] were determined using danpos.py script from DANPOS2 toolkit [52] with the default parameters in dpos mode. The reported fuzziness score was used as measurement of nucleosome positioning and subsequent filtering. Peak centers were annotated to genomic features (TSS, TTS and Exon/Intron boundary) using annotatePeaks.pl from HOMER package. Total peak count was normalized respectively by the occurrence of the feature. annotatePeaks.pl script was applied to calculate the nucleotide distribution of features and peaks as well.

### Nucleosome-nucleosome distance determination

The fragment size distribution of human as well as plasmodium derived di-nucleosomal fragments was estimated by a gaussian kernel density using fragments larger than 250 bp (SRX885811-SRX885819)[16]. The average nucleosome-nucleosome distance was inferred from the maximum of the kernel density estimate.

### Homology modeling of nucleosomal complexes

The standard protocol of YASARA [75] (version 16.4.6) was used to create homology models of all histones and the complete nucleosome consisting of an octamer that had 146bp of DNA wrapped around it. For each model of a nucleosomal complex, the input of YASARA was a multiple FASTA file with two DNA- and eight protein- sequences. The DNA-sequences were two copies of the palindromic DNA fragment (146bp long) from human X-chromosome alpha satellite DNA as found in PDB entry 3AFA. The protein-sequences were from the histones of *Homo sapiens* or *P*. *falciparum*, respectively. The GenBank accession numbers for the human histones were AAA63191.1 (H2A), AAN59961.1 (H2B), NP_066403.2 (H3), NP_003539.1 (H4) and for the plasmodial histones AAA29612.1 (H2A), XP_001347738.1 (H2B), AAO23910.1 (H3), AAP45785.1 (H4). Due to their flexibility, the N- and C-termini of histones could not be resolved in X-ray structures; thus, their 3D-orientation is unclear. This is why the histone sequences were trimmed according to the resolved 3D structure reported in PDB entry 3AFA.

In order to determine the homology models for plasmodium, three rounds of PSI-BLAST restricted to PDB entries [22] were conducted and YASARA selected PDB entries 3AFA, 5AV6, 3TU4, 3X1T and 2NQB as templates. These datasets represent the structures of nucleosomal core particles from different eukaryotic species. For the human template (3AFA), all of the 740 target residues could be aligned to template residues; for these the sequence identity was 84%. After building models for each template, YASARA combined the best scoring fragments of all models to deduce a hybrid homology model. The resulting hybrid model scored best and the internal quality assessment of YASARA determined an overall Z-score of0.056, which indicates model quality 0.056 standard deviations better than an average high-resolution X-ray structure. Note that model quality is most reliable for globular proteins and can be misleading for other protein types. Dihedrals and packing in 1D and 3D were rated as optimal by YASARA. The full models of a nucleosome in PDB-format can be found in the two Supplementary Files hu_complex.pdb (*Homo sapiens*) and pl_complex.pdb (*P*. *falciparum*).

### Molecular dynamics simulations

YASARA [24] was used to run three MD simulations for each nucleosome complex. In all runs, the complex was embedded into a water box; in order to vary experimental conditions, simulations were initiated with the different start-temperatures of 298.14 K, 298.15 K, and 298.16 K, respectively.

The data sets consisted of three MD trajectories each comprising 200 snapshots that represented varying poses of a 50 ns interval and FoldX [23] was used to calculate a score assessing the interaction energy. 200 snapshots with a time period of 250 ps were saved for subsequent processing; snapshots were stored in pdb format and contained the complex plus all water molecules within a maximal distance of 3 Å to a protein or DNA molecule. These snapshots were used to deduce mean values of scores assessing the following interactions, which were determined in a residue-specific manner: π-π stacking, cation-π stacking, contacts, hydrophobic interactions, and hydrogen-bond networks. For the first four interactions, scores were taken from the YASARA output; see YASARA documentation for details of computation. To score hydrogen-bond networks, distances were analyzed between residues, DNA, and water molecules in a snapshot-specific manner. Thus, a graph was computed that consisted of nodes that represent putatively interacting atoms on the surface of the considered molecules and of edges modelling hydrogen bonds. An edge was inserted, if the distance between a donor and an acceptor atom was not larger than 2.5 Å. Based on this network, a score was computed for each path interconnecting a pair of atoms from DNA and a protein according to:

$$S_{path}\left(atom_{i}^{k},\;atom_{j}^{l}\right)=1/\left(edges\left(atom_{i}^{k},\;atom_{j}^{l}\right)\;\#\;path\_ident\_len\right)$$ (1)

Here, $edges\left(atom_{i}^{k},\;atom_{j}^{l}\right)$ is the number of edges interconnecting an atom *k* of residue *I* with atom *l* of nucleotide *j* and the normalization factor #*path_ident_len* is the number of paths with the same length observed in the full data set. Thus, the score for a hydrogen-mediated interaction decreases with the number of involved water molecules and results in a higher score for a more direct one. The maximal number of co-operative water molecules was limited to one and for each residue *res*_*j*_, all *s*_*path*_-values were summed up.

For each of the averaged scores with noticeable amplitude, the log_2_-value was plotted for corresponding residues of the histones from *H*. *sapiens* and *P*. *falciparum* together with the sequences by means of a circos graph [76].

### Assessing the full interaction of histones and DNA

To analyze the interaction energy between individual histone cores and the DNA, FoldX was used (version 4, [23]). First, the side-chain orientation of all snapshots was optimized with the RepairPDB command to prepare the structures for the FoldX force-field. Then, mean interaction energies between Histone and DNA as well as their standard deviations were then deduced with the AnalyzeComplex command.

### Accession Numbers

The high-throughput sequencing data are available in the NCBI’s Sequence Read Archive (SRA) (accession: SRP055417, SRP026365, SRP001451, SRP001452).

## Supporting Information

S1 Fig A scoring of interaction-differences for nucleosomal residues. The outermost circle is an alignment of the residues from the four histones H2A, H2B, H3, and H4 from *H*. *sapiens* (outer sequence) and *P*. *falciparum* (inner sequence). Positions occupied by different residues are printed in red and additionally highlighted by a red rectangle in the innermost circle. The in-between circles consist of color-coded score values that indicate a higher score, if the color is dark. The order of the interactions is, if listed from the outside to the inside circles: hydrogen-bond networks, hydrophobic interactions, contacts, and cation-π stacking. Circle six summarizes the differences in a stack. Black boxes mark residues that possess at least one score belonging to the 10% most extreme values. No scores are given for the trimmed N- and C-termini.
(TIFF)

S2 Fig *P*. *falciparum* and human nucleosomes are positioned by the artificial nucleosome positioning sequence 601. The 208bp long 601-NotI DNA, harboring the 601 nucleosome positioning sequence at the border of the DNA fragment, was prepared by PCR and used for salt dialysis assembly of nucleosomes. 4μg of DNA were incubated with increasing amounts of the indicated histones (*P*. *falciparum*, lanes 1–4; human lanes 5 and 6) at high salt concentrations and dialyzed o/n. Nucleosome assembly and positions were analyzed by EMSA and stained with ethidium bromide. (*B*) Temperature induced nucleosome sliding. *P*. *falciparum* nucleosomes and human nucleosomes were separately reconstituted on Cy3 and Cy5 labelled KahrP DNA, amplified from the plasmodium genome and then mixed in equimolar ratios. Nucleosomal mixtures were incubated 60 min at room temperature (lane 1) or at the indicated temperatures (lanes 2 to 7). Nucleosome positions were analyzed after the temperature incubation on a native 5% polyacrylamide gel and visualized by fluorescence scanning (upper panel: cy5 labelled plasmodium nucleosomes; lower panel: cy3 labelled human nucleosomes). The positions of the nucleosomes and the temperature induced nucleosome position (open triangle) are indicated. (*C*) To analyze whether the full complement of histones is present on DNA, the 601-DNA was reconstituted into nucleosomes, incubated at elevated temperatures and separated on native polyacrylamide gels (lanes 1 and 2). Nucleosomes were excised from the gel after ethidium bromide staining and the gel pieces were equilibrated with Lämmli buffer, heated to 95°C and loaded on top of a 17% SDS-PAGE. The histone content of the nucleosome was analyzed after silver staining of the protein gel. The input of the human and plasmodium histones (lanes 3 and 5) is shown next to the protein content of the excised nucleosomal DNA (lanes 4 and 6).
(TIFF)

S3 Fig Determining the binding of histone octamers to AT-rich DNA with flanking GC-rich sequences. (*A*) A 147bp long AT-rich *P*. *falciparum* sequence originating from the KahrP promoter, with an AT content of 85,2% was mixed with DNA fragments having the same AT-core sequence (white rectangle), extended with either 71 and 32bp, or 32 and 26/16bp of GC-rich linker DNA. DNA fragments were mixed at equimolar ratios and used for nucleosome assembly with either human (lanes 2 and 3) or plasmodium histone octamers (lanes 4 and 5). Relative ratios of the individual DNA fragments did not change, showing that short, flanking GC-rich sequences are not sufficient to allow high affinity binding of the histones to GC-rich DNA. The asterisk marks a competitor DNA molecule (linearized pUC19) that is included to allow fine titration of the assembly reaction. The competitor is shifted to the well upon binding of histone octamers. Nucleosomes are analyzed on 6% polyacrylamide gels that allow the resolution of the free DNA. (*B*) Chloroquine stability assay. Scan of the Cy3 channel of the experiment shown in Fig 3B. The 210 bp KahrP DNA (AT-rich, Cy3-labelled, lane 13), either reconstituted with human (hs nucleosomes, lanes 1–6) or plasmodium octamers (pf nucleosomes, lanes 7–12) into nucleosomes is shown. Nucleosomes were incubated with increasing concentrations of chloroquine (0 to 9 mM), incubated for 10 min at room temperature and then analyzed by native polyacrylamide gel electrophoresis. The free DNA (AT-rich, lane 13) and contaminating single stranded DNA is indicated (asterisk).
(TIFF)

S4 Fig Plasmodium nucleosomes do preferentially occupy GC-rich DNA sequences. (*A*) Example locus of the *P*. *falciparum* genome located on chromosome 7. Genomic location and scale are indicated on top. Tracks show profiles of the nucleosome occupancy (red, top) and the nucleosome free regions determined by FAIRE-seq (cyan, middle) that were re-analyzed from datasets generated by Ponts and colleagues [14]. The average GC-content in a 50bp non-overlapping window (grey, bottom) is indicated below. Red and cyan boxes reveal computationally identified nucleosomes/nucleosome-free regions. (*B*) Kernel density plots of the GC content of computationally identified nucleosomes (red), nucleosome free (cyan) and randomly chosen genomic regions (grey). (*C*) Bar graphs representing the fraction of identified nucleosomes in exons (red), introns (blue) and intergenic regions (green), compared to their genome wide occurrence (top). The averaged GC contents of exons, introns and intergenic regions of the *P*. *falciparum* genome are indicated at the bottom. (*D*) Kernel density plot of the GC content of computationally identified nucleosomes. Nucleosome occupancy datasets (SRX885811-SRX885819) generated by Kensche and colleagues were re-analyzed and nucleosome positions were predicted using DANPOS2 as described in the material and methods section. (E) Scatter plot illustrating the correlation of GC-content with nucleosome occupancy of computationally identified nucleosomes (based on datasets generated by Kensche and colleagues). Data points with a cooks distance > 0.0001 were excluded. The relationship between GC-content and nucleosome occupancy has been modelled using a linear regression analysis.
(TIFF)

S5 Fig The amino acid exchange in the histone H4 tail affects the SNF2H dependent nucleosome remodeling kinetics of *P*. *falciparum* nucleosomes. (*A*) Competitive electromobility shift assays with Cy5-labelled plasmodium nucleosomes (left panel) and Cy3-labelled human nucleosomes (middle panel) reconstituted on 601 DNA. Like in the chromatin remodeling assay, plasmodium and human nucleosomes were mixed at equimolar ratios and incubated with increasing concentrations of SNF2H. However, the electromobility shift reactions were incubated for 10 min at room temperature in the absence of ATP and no competitor DNA was added prior to electrophoresis. The gel was stained by fluorescence imaging (cy5, representing the plasmodium nucleosomes, lanes 1–5; cy3, representing the human nucleososomes, lanes 6–10) and subsequently with ethidium bromide (lanes 11–15). The position of the free DNA and nucleosomes are indicated. (*B*) Scheme showing the sequence differences of the plasmodium and human histone H4 N-terminus. The RHR box indicates the residues that were previously identified to affect SNF2H ATPase activity. The V to I amino acid change at position 21 is marked by an X. (*C*) The H4hyb octamers consisting of the plasmodium histones H2A, H2B, H3 and the human histone H4 were reconstituted into nucleosomes and assayed SNF2H dependent nucleosome remodeling (cy5 upper panel) in the presence of equimolar amounts of human nucleosomes (cy3 lower panel). Reactions were incubated for 60 min at 37°C in the presence or absence of ATP, as indicated. Nucleosome positions were analyzed by EMSA and imaged for the Cy5 and Cy3 channel, respectively. The positions of the nucleosomes are indicated on the right side and an asterisk marks a non-specific band.
(TIFF)

S6 Fig The nucleosomal core DNA of plasmodium nucleosomes exhibits resistance towards MNase dependent hydrolysis. (A) Recombinant plasmodium histone octamers were used for chromatin assembly with a 11kb plasmid by salt dialysis. Chromatin was subjected to partial MNase digestion (10u; 20 to 125 sec), stopping the reaction by the addition of SDS/EDTA. DNA was purified and visualized by agarose gel electrophoresis and ethidium bromide staining. The position of the mono-nucleosomal DNA is indicated (<nuc.). (B) In order to reveal the size of the MNase hydrolysis products in high resolution, samples were separated on the Agilent TapeStation using the DNA1000 kit. Samples shown in (A) (lanes 2–4) and an additional MNase hydrolysis reaction using the twofold amount of MNase (20u) and increased hydrolysis times (20 to 640 sec; lanes 5–10) were analyzed on the TapeStation. The TapeStation analysis software was used to determine the length of the protected DNA fragment and plotted below (median size of nucleosome protected DNA fragment; red). The positions of the protected nucleosomal DNA fragment and the internal system markers are indicated. (C) DNA length distribution plots revealing the MNase protected nucleosomal DNA lengths *in vivo*. The size distribution of mono-nucleosomal DNA fragments derived from human (red) as well as plasmodium DNA (black) after MNase digestions (SRX885811-SRX885819)[16]. MNase digestions were performed at different stages of the erythrocytic life cycle of *P*. *falciparum*. Each stage of the life cycle is plotted individually together with the corresponding human DNA fraction.
(TIFF)

S7 Fig *P*. *falciparum* histone octamers display different binding characteristics towards sequence dependent nucleosome positioning signals. (A) A 6% native gel showing the free DNA used for nucleosome assembly reactions, presented in Fig 5C. The different DNA templates have been prepared by restriction enzyme digestion and purification from a plasmid containing the genomic region from position 1307500–1308399 on chromosome 11. (*B*) Di-nucleotide frequency of nucleosome core fragments derived from human (left) and plasmodium (right) nucleosomal DNA. Data was extracted from the study performed by the LeRoch laboratories [15]. Position-dependent frequencies of A/T (AA/TT/AT/TA) and C/G (CC/GG/CG/GC) di-nucleotides are illustrated. (*C*) The same analysis as shown in (B), was performed with the dataset taken from the publication of Kensche and colleagues [16]. Position-dependent frequencies of A/T (AA/TT/AT/TA) and C/G (CC/GG/CG/GC) di-nucleotides are illustrated. The positions of histone-DNA contact points, the superhelical loop locations (SHL), are shown by dotted blue lines and annotated.
(TIFF)

S8 Fig AT-repeat sequences in the linker region of the nucleosome determine the positioning of plasmodial histone octamers. (*A*) Nucleosome positioning at the beginning of the coding region (ATG), the end of the coding region (ECR) and the exon/intron boundaries of all annotated *P*. *falciparum* genes were aligned based on the datasets of Kensche and colleagues [16]. Individual nucleosome positions were further characterized by a fuzziness score and the average fuzziness signal for all aligned genes was plotted as a heatmap (top). The color code is indicated on the right. The average frequency of identified nucleosome midpoints (middle) and of AT/TA (red) and AA/TT di-nucleotides (blue) over regulatory regions of all annotated genes is given. (*B*) Changes in AT/TA and AA/TT di-nucleotide frequencies within the nucleosome and its linker regions compared to the fuzziness score of nucleosome positioning. Five different fractions of nucleosomes, from well positioned (dark blue) to badly positioned (light blue) nucleosomes for *P*. *falciparum* (top panel) and *S*. *cerevisiae* (bottom panel) were analyzed. Vertical lines indicate nucleosome boundaries. The color code for the fuzziness score is indicated below. (*C*) The sequence used to analyze nucleosome positioning in Fig 6C was chosen according to its potential to form positioned nucleosomes *in vivo*. As a measure for nucleosome positioning we used the ChIP-Seq experiments performed by the Stunnenberg lab [77] that are indicated in the screen shot of the genome browser (lower panel). According to the data, we synthesized a 300bp long DNA fragment, representing the *P*. *falciparum* sequence from position 1.307.900 to 1.308.200 of chromosome 11 (Pf3D7 v3). The AT-content of this sequence is 85.2%.
(TIFF)

## References

[1] Gardner MJ, Hall N, Fung E, White O, Berriman M, Hyman RW, et al Genome sequence of the human malaria parasite Plasmodium falciparum. Nature. 2002;419: 498–511. 10.1038/nature01097 12368864 PMC3836256

[2] Bozdech Z, Llinás M, Pulliam BL, Wong ED, Zhu J, DeRisi JL. The transcriptome of the intraerythrocytic developmental cycle of Plasmodium falciparum. PLoS Biol. 2003;1: E5 10.1371/journal.pbio.0000005 12929205 PMC176545

[3] Le Roch KG, Zhou Y, Blair PL, Grainger M, Moch JK, Haynes JD, et al Discovery of gene function by expression profiling of the malaria parasite life cycle. Science. American Association for the Advancement of Science; 2003;301: 1503–1508. 10.1126/science.1087025 12893887

[4] Templeton TJ, Iyer LM, Anantharaman V, Enomoto S, Abrahante JE, Subramanian GM, et al Comparative analysis of apicomplexa and genomic diversity in eukaryotes. Genome Res. Cold Spring Harbor Lab; 2004;14: 1686–1695. 10.1101/gr.2615304 PMC515313 15342554

[5] Luger K, Mäder AW, Richmond RK, Sargent DF, Richmond TJ. Crystal structure of the nucleosome core particle at 2.8 A resolution. Nature. 1997;389: 251–260. 10.1038/38444 9305837

[6] Malik HS, Henikoff S. Phylogenomics of the nucleosome. Nat Struct Biol. 2003;10: 882–891. 10.1038/nsb996 14583738

[7] Lohr D, Corden J, Tatchell K, Kovacic RT, van Holde KE. Comparative subunit structure of HeLa, yeast, and chicken erythrocyte chromatin. Proc Natl Acad Sci USA. 1977;74: 79–83. 319461 10.1073/pnas.74.1.79 PMC393200

[8] Lowary PT, Widom J. New DNA sequence rules for high affinity binding to histone octamer and sequence-directed nucleosome positioning. J Mol Biol. 1998;276: 19–42. 10.1006/jmbi.1997.1494 9514715

[9] Segal E, Fondufe-Mittendorf Y, Chen L, Thåström A, Field Y, Moore IK, et al A genomic code for nucleosome positioning. Nature. 2006;442: 772–778. 10.1038/nature04979 16862119 PMC2623244

[10] Horrocks P, Lanzer M. Differences in nucleosome organization over episomally located plasmids coincides with aberrant promoter activity in P. falciparum. Parasitol Int. 1999;48: 55–61. 11269326 10.1016/s1383-5769(99)00002-1

[11] Horrocks P, Pinches R, Kriek N, Newbold C. Stage-specific promoter activity from stably maintained episomes in Plasmodium falciparum. Int J Parasitol. 2002;32: 1203–1206. 12204219 10.1016/s0020-7519(02)00123-6

[12] Duraisingh MT, Voss T, Marty AJ, Duffy MF, Good RT, Thompson JK, et al Heterochromatin silencing and locus repositioning linked to regulation of virulence genes in Plasmodium falciparum. Cell. Elsevier; 2005;121: 13–24. 10.1016/j.cell.2005.01.036 15820675

[13] Freitas LH Junior, Hernandez-Rivas R, Ralph SA, Montiel-Condado D, Ruvalcaba-Salazar OK, Rojas-Meza AP, et al Telomeric heterochromatin propagation and histone acetylation control mutually exclusive expression of antigenic variation genes in malaria parasites. Cell. 2005;121: 25–36. 10.1016/j.cell.2005.01.037 15820676

[14] Ponts N, Harris EY, Prudhomme J, Wick I, Eckhardt-Ludka C, Hicks GR, et al Nucleosome landscape and control of transcription in the human malaria parasite. Genome Res. 2010;20: 228–238. 10.1101/gr.101063.109 20054063 PMC2813478

[15] Bunnik EM, Polishko A, Prudhomme J, Ponts N, Gill SS, Lonardi S, et al DNA-encoded nucleosome occupancy is associated with transcription levels in the human malaria parasite Plasmodium falciparum. BMC Genomics. 2014;15: 347 10.1186/1471-2164-15-347 24885191 PMC4035074

[16] Kensche PR, Hoeijmakers WAM, Toenhake CG, Bras M, Chappell L, Berriman M, et al The nucleosome landscape of Plasmodium falciparum reveals chromatin architecture and dynamics of regulatory sequences. Nucleic Acids Res. 2016;44: 2110–2124. 10.1093/nar/gkv1214 26578577 PMC4797266

[17] Westenberger SJ, Cui L, Dharia N, Winzeler E, Cui L. Genome-wide nucleosome mapping of Plasmodium falciparum reveals histone-rich coding and histone-poor intergenic regions and chromatin remodeling of core and subtelomeric genes. BMC Genomics. 2009;10: 610 10.1186/1471-2164-10-610 20015349 PMC2801526

[18] Tillo D, Hughes TR, Hughes TR. G+C content dominates intrinsic nucleosome occupancy. BMC Bioinformatics. 2009;10: 442 10.1186/1471-2105-10-442 20028554 PMC2808325

[19] Segal E, Widom J. Poly(dA:dT) tracts: major determinants of nucleosome organization. Curr Opin Struct Biol. 2009;19: 65–71. 10.1016/j.sbi.2009.01.004 19208466 PMC2673466

[20] Baxevanis AD, Landsman D. Histone Sequence Database: new histone fold family members. Nucleic Acids Res. 1998;26: 372–375. 9399877 10.1093/nar/26.1.372 PMC147196

[21] Luger K, Rechsteiner TJ, Flaus A, Waye MM, Richmond TJ. Characterization of nucleosome core particles containing histone proteins made in bacteria. J Mol Biol. 1997;272: 301–311. 10.1006/jmbi.1997.1235 9325091

[22] Bernstein FC, Koetzle TF, Williams GJ, Meyer EF, Brice MD, Rodgers JR, et al The Protein Data Bank. A computer-based archival file for macromolecular structures. Eur J Biochem. 1977;80: 319–324. 923582 10.1111/j.1432-1033.1977.tb11885.x

[23] Guerois R, Nielsen JE, Serrano L. Predicting changes in the stability of proteins and protein complexes: a study of more than 1000 mutations. J Mol Biol. 2002;320: 369–387. 10.1016/S0022-2836(02)00442-4 12079393

[24] Krieger E, Darden T, Nabuurs SB, Finkelstein A, Vriend G. Making optimal use of empirical energy functions: force-field parameterization in crystal space. Proteins. Wiley Subscription Services, Inc., A Wiley Company; 2004;57: 678–683. 10.1002/prot.20251 15390263

[25] Flaus A, Owen-Hughes TA. Dynamic properties of nucleosomes during thermal and ATP-driven mobilization. Mol Cell Biol. 2003;23: 7767–7779. 10.1128/MCB.23.21.7767-7779.2003 14560021 PMC207611

[26] Meersseman G, Pennings S, Bradbury EM. Mobile nucleosomes—a general behavior. EMBO J. 1992;11: 2951–2959. 1639066 10.1002/j.1460-2075.1992.tb05365.x PMC556777

[27] Flaus A, Richmond TJ. Positioning and stability of nucleosomes on MMTV 3'LTR sequences. J Mol Biol. 1998;275: 427–441. 10.1006/jmbi.1997.1464 9466921

[28] Thåström A, Lowary PT, Widlund HR, Cao H, Kubista M, Widom J. Sequence motifs and free energies of selected natural and non-natural nucleosome positioning DNA sequences. J Mol Biol. 1999;288: 213–229. 10.1006/jmbi.1999.2686 10329138

[29] Hamiche A, Sandaltzopoulos R, Gdula DA, Wu C. ATP-dependent histone octamer sliding mediated by the chromatin remodeling complex NURF. Cell. 1999;97: 833–842. 10399912 10.1016/s0092-8674(00)80796-5

[30] Schröter H, Maier G, Ponstingl H, Nordheim A. DNA intercalators induce specific release of HMG 14, HMG 17 and other DNA-binding proteins from chicken erythrocyte chromatin. EMBO J. 1985;4: 3867–3872. 4092697 10.1002/j.1460-2075.1985.tb04159.x PMC554742

[31] Burton DR, Butler MJ, Hyde JE, Phillips D, Skidmore CJ, Walker IO. The interaction of core histones with DNA: equilibrium binding studies. Nucleic Acids Res. 1978;5: 3643–3663. 724497 10.1093/nar/5.10.3643 PMC342701

[32] Yager TD, McMurray CT, van Holde KE. Salt-induced release of DNA from nucleosome core particles. Biochemistry. 1989;28: 2271–2281. 2719953 10.1021/bi00431a045

[33] Bao Y, White CL, Luger K. Nucleosome core particles containing a poly(dA.dT) sequence element exhibit a locally distorted DNA structure. J Mol Biol. 2006;361: 617–624. 10.1016/j.jmb.2006.06.051 16860337

[34] Anderson JD, Widom J. Poly(dA-dT) promoter elements increase the equilibrium accessibility of nucleosomal DNA target sites. Mol Cell Biol. 2001;21: 3830–3839. 10.1128/MCB.21.11.3830-3839.2001 11340174 PMC87046

[35] Nelson HC, Finch JT, Luisi BF, Klug A. The structure of an oligo(dA).oligo(dT) tract and its biological implications. Nature. Nature Publishing Group; 1987;330: 221–226. 10.1038/330221a0 3670410

[36] Satchwell SC, Drew HR, Travers AA. Sequence periodicities in chicken nucleosome core DNA. J Mol Biol. 1986;191: 659–675. 3806678 10.1016/0022-2836(86)90452-3

[37] Tillo D, Kaplan N, Moore IK, Moore IK, Fondufe-Mittendorf Y, Fondufe-Mittendorf Y, et al High nucleosome occupancy is encoded at human regulatory sequences. Hannenhalli S, editor. PLoS ONE. 2010;5: e9129 10.1371/journal.pone.0009129 20161746 PMC2817738

[38] Widom J. Role of DNA sequence in nucleosome stability and dynamics. Q Rev Biophys. 2002;34: 269–324. 10.1017/s0033583501003699 11838235

[39] Giresi PG, Kim J, McDaniell RM, Iyer VR, Lieb JD. FAIRE (Formaldehyde-Assisted Isolation of Regulatory Elements) isolates active regulatory elements from human chromatin. Genome Res. 2007;17: 877–885. 10.1101/gr.5533506 17179217 PMC1891346

[40] ROBERTS WK, DEKKER CA, RUSHIZKY GW, KNIGHT CA. Studies on the mechanism of action of micrococcal nuclease. 1. Degradation of thymus deoxyribonucleic acid. Biochim Biophys Acta. 1962;55: 664–673. 14492805 10.1016/0006-3002(62)90844-2

[41] Längst G, Bonte EJ, Corona DFV, Becker PB. Nucleosome movement by CHRAC and ISWI without disruption or trans-displacement of the histone octamer. Cell. 1999;97: 843–852. 10399913 10.1016/s0092-8674(00)80797-7

[42] Erdel F, Krug J, Längst G, Rippe K. Targeting chromatin remodelers: signals and search mechanisms. Biochim Biophys Acta. 2011;1809: 497–508. 10.1016/j.bbagrm.2011.06.005 21704204

[43] Clapier CR, Längst G, Corona DFV, Becker PB, Nightingale KP. Critical role for the histone H4 N terminus in nucleosome remodeling by ISWI. Mol Cell Biol. 2001;21: 875–883. 10.1128/MCB.21.3.875-883.2001 11154274 PMC86678

[44] Clapier CR, Nightingale KP, Becker PB. A critical epitope for substrate recognition by the nucleosome remodeling ATPase ISWI. Nucleic Acids Res. 2002;30: 649–655. 11809876 10.1093/nar/30.3.649 PMC100309

[45] Woodcock CL, Ghosh RP. Chromatin higher-order structure and dynamics. Cold Spring Harb Perspect Biol. 2010;2: a000596 10.1101/cshperspect.a000596 20452954 PMC2857170

[46] Lanzer M, Wertheimer SP, de Bruin D, Ravetch JV. Chromatin structure determines the sites of chromosome breakages in Plasmodium falciparum. Nucleic Acids Res. 1994;22: 3099–3103. 8065922 10.1093/nar/22.15.3099 PMC310281

[47] Luger K, Dechassa ML, Tremethick DJ. New insights into nucleosome and chromatin structure: an ordered state or a disordered affair? Nat Rev Mol Cell Biol. 2012;13: 436–447. 10.1038/nrm3382 22722606 PMC3408961

[48] Ioshikhes I, Bolshoy A, Derenshteyn K, Borodovsky M, Trifonov EN. Nucleosome DNA sequence pattern revealed by multiple alignment of experimentally mapped sequences. J Mol Biol. 1996;262: 129–139. 10.1006/jmbi.1996.0503 8831784

[49] Rippe K, Schrader A, Riede P, Strohner R, Lehmann E, Längst G. DNA sequence- and conformation-directed positioning of nucleosomes by chromatin-remodeling complexes. Proc Natl Acad Sci USA. National Acad Sciences; 2007;104: 15635–15640. 10.1073/pnas.0702430104 PMC2000439 17893337

[50] Ay F, Bunnik EM, Varoquaux N, Bol SM, Prudhomme J, Vert J-P, et al Three-dimensional modeling of the P. falciparum genome during the erythrocytic cycle reveals a strong connection between genome architecture and gene expression. Genome Res. Cold Spring Harbor Lab; 2014;24: 974–988. 10.1101/gr.169417.113 PMC4032861 24671853

[51] Brogaard K, Xi L, Wang J-P, Widom J. A map of nucleosome positions in yeast at base-pair resolution. Nature. 2012;486: 496–501. 10.1038/nature11142 22722846 PMC3786739

[52] Chen K, Xi Y, Pan X, Li Z, Kaestner K, Tyler J, et al DANPOS: dynamic analysis of nucleosome position and occupancy by sequencing. Genome Res. Cold Spring Harbor Lab; 2013;23: 341–351. 10.1101/gr.142067.112 PMC3561875 23193179

[53] Cary C, Lamont D, Dalton JP, Doerig C. Plasmodium falciparum chromatin: nucleosomal organisation and histone-like proteins. Parasitol Res. 1994;80: 255–258. 8036241 10.1007/BF00932684

[54] Correll SJ, Schubert MH, Grigoryev SA. Short nucleosome repeats impose rotational modulations on chromatin fibre folding. EMBO J. 2012;31: 2416–2426. 10.1038/emboj.2012.80 22473209 PMC3364735

[55] Perišić O, Collepardo-Guevara R, Schlick T. Modeling studies of chromatin fiber structure as a function of DNA linker length. J Mol Biol. 2010;403: 777–802. 10.1016/j.jmb.2010.07.057 20709077 PMC2966533

[56] Kruger W, Peterson CL, Sil A, Coburn C, Arents G, Moudrianakis EN, et al Amino acid substitutions in the structured domains of histones H3 and H4 partially relieve the requirement of the yeast SWI/SNF complex for transcription. Genes Dev. 1995;9: 2770–2779. 7590252 10.1101/gad.9.22.2770

[57] Fleming AB, Pennings S. Antagonistic remodelling by Swi-Snf and Tup1-Ssn6 of an extensive chromatin region forms the background for FLO1 gene regulation. EMBO J. 2001;20: 5219–5231. 10.1093/emboj/20.18.5219 11566885 PMC125633

[58] Wechser MA, Kladde MP, Alfieri JA, Peterson CL. Effects of Sin- versions of histone H4 on yeast chromatin structure and function. EMBO J. 1997;16: 2086–2095. 10.1093/emboj/16.8.2086 9155034 PMC1169811

[59] Kurumizaka H, Wolffe AP. Sin mutations of histone H3: influence on nucleosome core structure and function. Mol Cell Biol. 1997;17: 6953–6969. 9372928 10.1128/mcb.17.12.6953 PMC232553

[60] Luger K. Structure and dynamic behavior of nucleosomes. Curr Opin Genet Dev. 2003;13: 127–135. 12672489 10.1016/s0959-437x(03)00026-1

[61] Lohr D, Tatchell K, van Holde KE. On the occurrence of nucleosome phasing in chromatin. Cell. 1977;12: 829–836. 336221 10.1016/0092-8674(77)90281-1

[62] Sussman JL, Trifonov EN. Possibility of nonkinked packing of DNA in chromatin. Proc Natl Acad Sci USA. National Academy of Sciences; 1978;75: 103–107. 10.1073/pnas.75.1.103 PMC411192 272625

[63] Fraser RM, Keszenman-Pereyra D, Simmen MW, Allan J. High-resolution mapping of sequence-directed nucleosome positioning on genomic DNA. J Mol Biol. 2009;390: 292–305. 10.1016/j.jmb.2009.04.079 19427325

[64] Kogan SB, Kato M, Kiyama R, Trifonov EN. Sequence structure of human nucleosome DNA. J Biomol Struct Dyn. 2006;24: 43–48. 10.1080/07391102.2006.10507097 16780374

[65] Korber P. Active nucleosome positioning beyond intrinsic biophysics is revealed by in vitro reconstitution. Biochem Soc Trans. 2012;40: 377–382. 10.1042/BST20110730 22435815

[66] Cui F, Zhurkin VB. Structure-based analysis of DNA sequence patterns guiding nucleosome positioning in vitro. J Biomol Struct Dyn. 2010;27: 821–841. 10.1080/073911010010524947 20232936 PMC2993692

[67] Lieleg C, Krietenstein N, Walker M, Korber P. Nucleosome positioning in yeasts: methods, maps, and mechanisms. Chromosoma. 2014. 10.1007/s00412-014-0501-x 25529773

[68] Luger K, Rechsteiner TJ, Richmond TJ. Expression and purification of recombinant histones and nucleosome reconstitution. Methods Mol Biol. New Jersey: Humana Press; 1999;119: 1–16. 10.1385/1-59259-681-9:1 10804500

[69] Rhodes D, Laskey RA. Assembly of nucleosomes and chromatin in vitro. Meth Enzymol. 1989;170: 575–585. 2770552 10.1016/0076-6879(89)70065-3

[70] Flaus A, Owen-Hughes TA. Mechanisms for nucleosome mobilization. Biopolymers. Wiley Subscription Services, Inc., A Wiley Company; 2003;68: 563–578. 10.1002/bip.10323 12666181

[71] Langmead B, Salzberg SL. Fast gapped-read alignment with Bowtie 2. Nat Meth. 2012;9: 357–359. 10.1038/nmeth.1923 PMC3322381 22388286

[72] Li H, Handsaker B, Wysoker A, Fennell T, Ruan J, Homer N, et al The Sequence Alignment/Map format and SAMtools. Bioinformatics. 2009;25: 2078–2079. 10.1093/bioinformatics/btp352 19505943 PMC2723002

[73] Quinlan AR, Hall IM. BEDTools: a flexible suite of utilities for comparing genomic features. Bioinformatics. Oxford University Press; 2010;26: 841–842. 10.1093/bioinformatics/btq033 PMC2832824 20110278

[74] Heinz S, Benner C, Spann N, Bertolino E, Lin YC, Laslo P, et al Simple combinations of lineage-determining transcription factors prime cis-regulatory elements required for macrophage and B cell identities. 2010;38: 576–589. 10.1016/j.molcel.2010.05.004 PMC2898526 20513432

[75] Krieger E, Joo K, Lee J, Lee J, Raman S, Thompson J, et al Improving physical realism, stereochemistry, and side-chain accuracy in homology modeling: Four approaches that performed well in CASP8. Proteins. Wiley Subscription Services, Inc., A Wiley Company; 2009;77 Suppl 9: 114–122. 10.1002/prot.22570 PMC2922016 19768677

[76] Krzywinski M, Schein J, Birol I, Connors J, Gascoyne R, Horsman D, et al Circos: an information aesthetic for comparative genomics. Genome Res. Cold Spring Harbor Lab; 2009;19: 1639–1645. 10.1101/gr.092759.109 PMC2752132 19541911

[77] Bártfai R, Hoeijmakers WAM, Salcedo-Amaya AM, Smits AH, Janssen-Megens E, Kaan A, et al H2A.Z demarcates intergenic regions of the plasmodium falciparum epigenome that are dynamically marked by H3K9ac and H3K4me3. PLoS Pathog. 2010;6: e1001223 10.1371/journal.ppat.1001223 21187892 PMC3002978

