# Supplementary figures and images for: Plasmodium falciparum Nucleosomes Exhibit Reduced Stability and Lost Sequence Dependent Nucleosome Positioning
Source: PLoS Pathog. 2016 Dec 29;12(12):e1006080. doi: 10.1371/journal.ppat.1006080 (PMC5198986; doi:10.1371/journal.ppat.1006080)

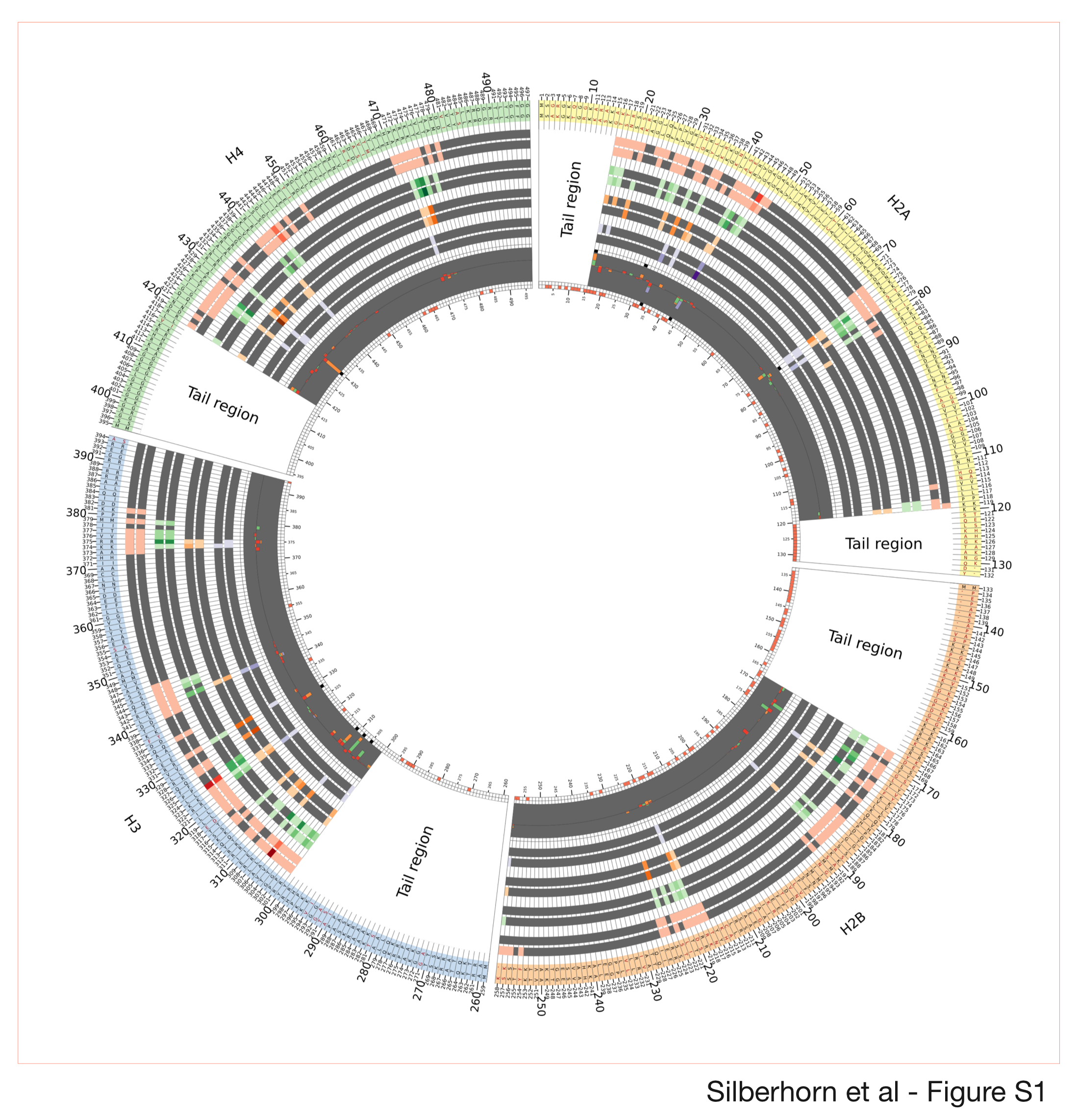

Supplement: S1 Fig — The outermost circle is an alignment of the residues from the four histones H2A, H2B, H3, and H4 from H. sapiens (outer sequence) and P. falciparum (inner sequence). Positions occupied by different residues are printed in red and additionally highlighted by a red rectangle in the innermost circle. The in-between circles consist of color-coded score values that indicate a higher score, if the color is dark. The order of the interactions is, if listed from the outside to the inside circles: hydrogen-bond networks, hydrophobic interactions, contacts, and cation-π stacking. Circle six summarizes the differences in a stack. Black boxes mark residues that possess at least one score belonging to the 10% most extreme values. No scores are given for the trimmed N- and C-termini. (TIFF) [file ppat.1006080.s001.tiff]

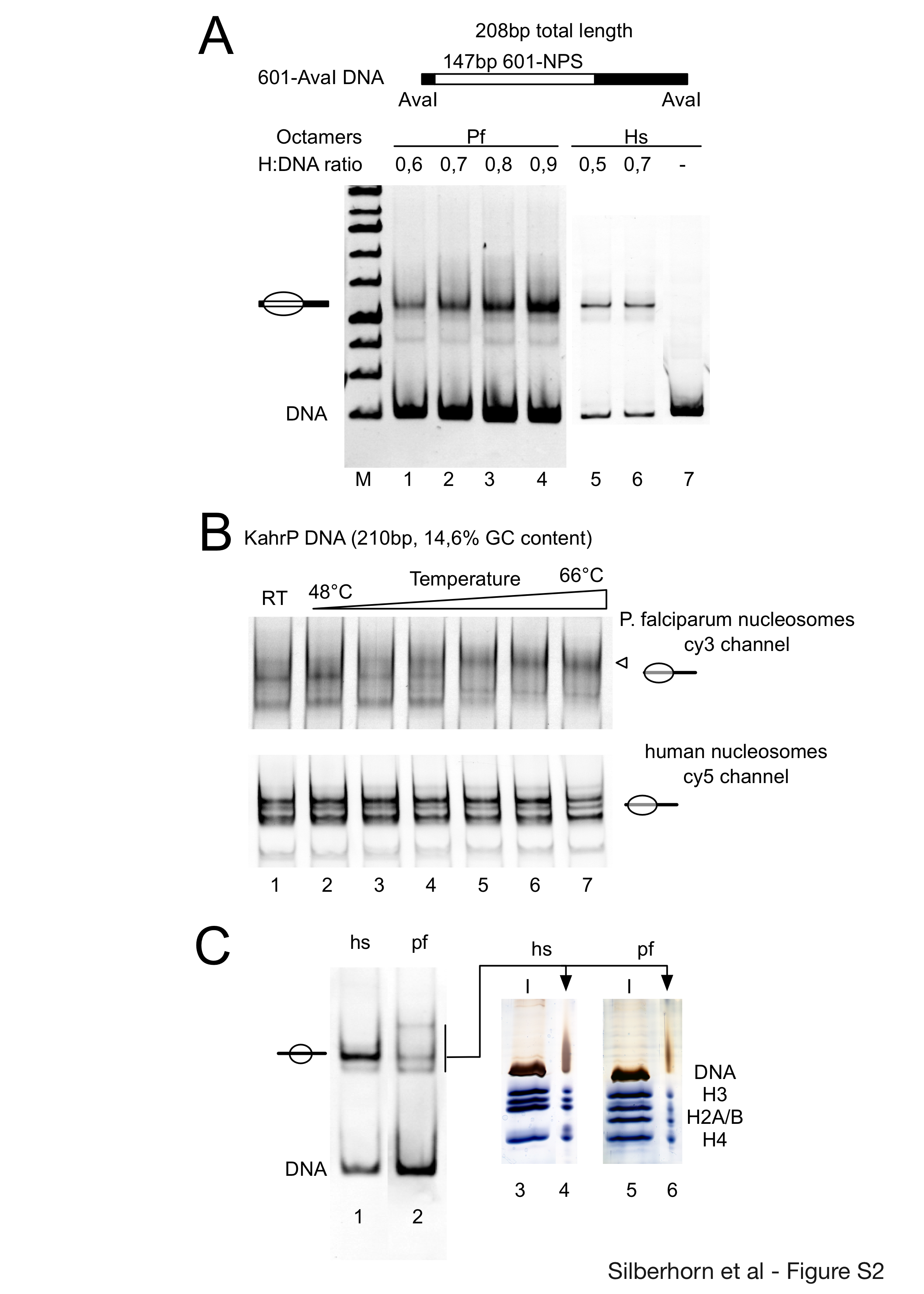

Supplement: S2 Fig — The 208bp long 601-NotI DNA, harboring the 601 nucleosome positioning sequence at the border of the DNA fragment, was prepared by PCR and used for salt dialysis assembly of nucleosomes. 4μg of DNA were incubated with increasing amounts of the indicated histones (P. falciparum, lanes 1–4; human lanes 5 and 6) at high salt concentrations and dialyzed o/n. Nucleosome assembly and positions were analyzed by EMSA and stained with ethidium bromide. (B) Temperature induced nucleosome sliding. P. falciparum nucleosomes and human nucleosomes were separately reconstituted on Cy3 and Cy5 labelled KahrP DNA, amplified from the plasmodium genome and then mixed in equimolar ratios. Nucleosomal mixtures were incubated 60 min at room temperature (lane 1) or at the indicated temperatures (lanes 2 to 7). Nucleosome positions were analyzed after the temperature incubation on a native 5% polyacrylamide gel and visualized by fluorescence scanning (upper panel: cy5 labelled plasmodium nucleosomes; lower panel: cy3 labelled human nucleosomes). The positions of the nucleosomes and the temperature induced nucleosome position (open triangle) are indicated. (C) To analyze whether the full complement of histones is present on DNA, the 601-DNA was reconstituted into nucleosomes, incubated at elevated temperatures and separated on native polyacrylamide gels (lanes 1 and 2). Nucleosomes were excised from the gel after ethidium bromide staining and the gel pieces were equilibrated with Lämmli buffer, heated to 95°C and loaded on top of a 17% SDS-PAGE. The histone content of the nucleosome was analyzed after silver staining of the protein gel. The input of the human and plasmodium histones (lanes 3 and 5) is shown next to the protein content of the excised nucleosomal DNA (lanes 4 and 6). (TIFF) [file ppat.1006080.s002.tiff]

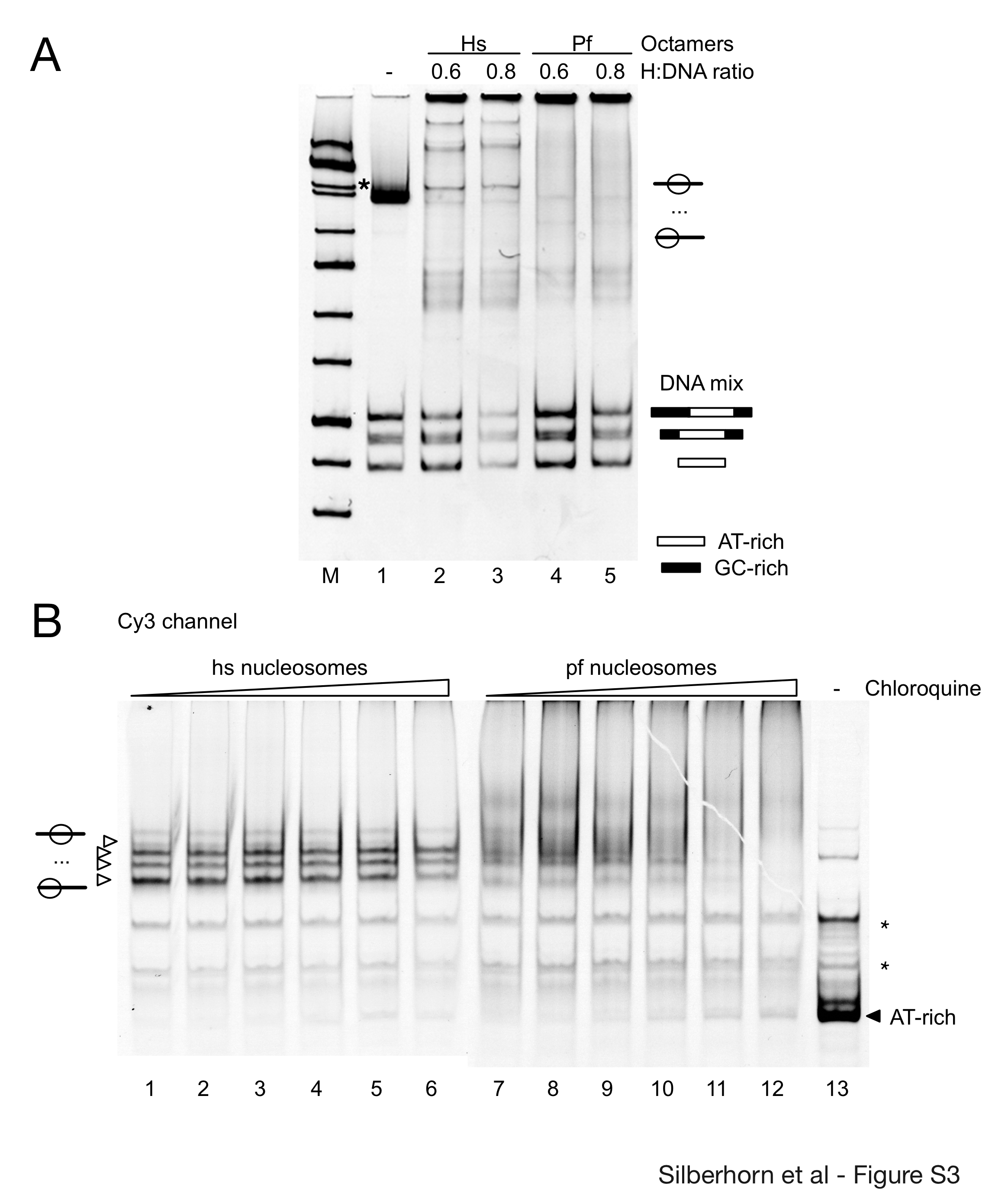

Supplement: S3 Fig — (A) A 147bp long AT-rich P. falciparum sequence originating from the KahrP promoter, with an AT content of 85,2% was mixed with DNA fragments having the same AT-core sequence (white rectangle), extended with either 71 and 32bp, or 32 and 26/16bp of GC-rich linker DNA. DNA fragments were mixed at equimolar ratios and used for nucleosome assembly with either human (lanes 2 and 3) or plasmodium histone octamers (lanes 4 and 5). Relative ratios of the individual DNA fragments did not change, showing that short, flanking GC-rich sequences are not sufficient to allow high affinity binding of the histones to GC-rich DNA. The asterisk marks a competitor DNA molecule (linearized pUC19) that is included to allow fine titration of the assembly reaction. The competitor is shifted to the well upon binding of histone octamers. Nucleosomes are analyzed on 6% polyacrylamide gels that allow the resolution of the free DNA. (B) Chloroquine stability assay. Scan of the Cy3 channel of the experiment shown in Fig 3B. The 210 bp KahrP DNA (AT-rich, Cy3-labelled, lane 13), either reconstituted with human (hs nucleosomes, lanes 1–6) or plasmodium octamers (pf nucleosomes, lanes 7–12) into nucleosomes is shown. Nucleosomes were incubated with increasing concentrations of chloroquine (0 to 9 mM), incubated for 10 min at room temperature and then analyzed by native polyacrylamide gel electrophoresis. The free DNA (AT-rich, lane 13) and contaminating single stranded DNA is indicated (asterisk). (TIFF) [file ppat.1006080.s003.tiff]

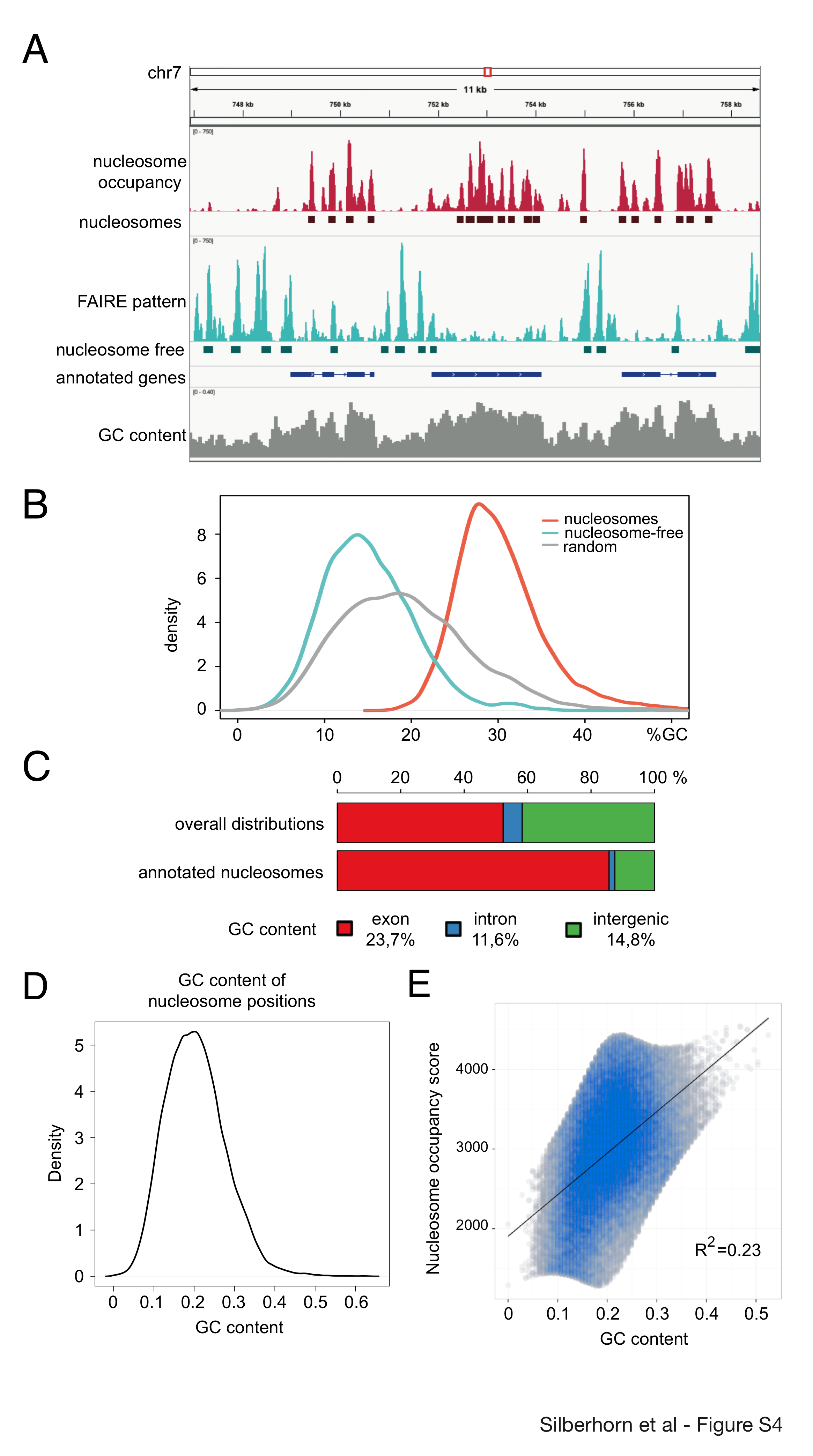

Supplement: S4 Fig — (A) Example locus of the P. falciparum genome located on chromosome 7. Genomic location and scale are indicated on top. Tracks show profiles of the nucleosome occupancy (red, top) and the nucleosome free regions determined by FAIRE-seq (cyan, middle) that were re-analyzed from datasets generated by Ponts and colleagues [14]. The average GC-content in a 50bp non-overlapping window (grey, bottom) is indicated below. Red and cyan boxes reveal computationally identified nucleosomes/nucleosome-free regions. (B) Kernel density plots of the GC content of computationally identified nucleosomes (red), nucleosome free (cyan) and randomly chosen genomic regions (grey). (C) Bar graphs representing the fraction of identified nucleosomes in exons (red), introns (blue) and intergenic regions (green), compared to their genome wide occurrence (top). The averaged GC contents of exons, introns and intergenic regions of the P. falciparum genome are indicated at the bottom. (D) Kernel density plot of the GC content of computationally identified nucleosomes. Nucleosome occupancy datasets (SRX885811-SRX885819) generated by Kensche and colleagues were re-analyzed and nucleosome positions were predicted using DANPOS2 as described in the material and methods section. (E) Scatter plot illustrating the correlation of GC-content with nucleosome occupancy of computationally identified nucleosomes (based on datasets generated by Kensche and colleagues). Data points with a cooks distance > 0.0001 were excluded. The relationship between GC-content and nucleosome occupancy has been modelled using a linear regression analysis. (TIFF) [file ppat.1006080.s004.tiff]

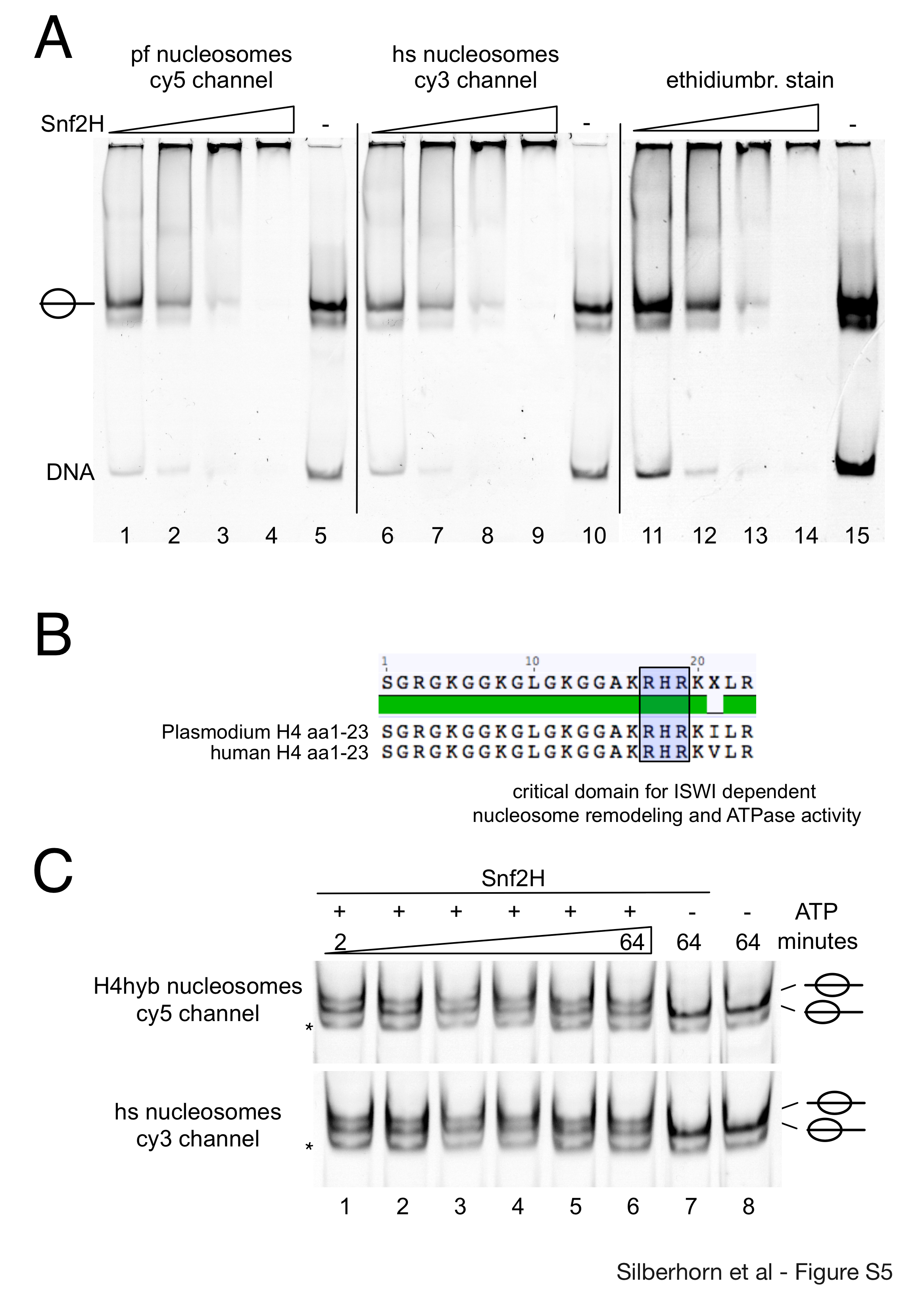

Supplement: S5 Fig — (A) Competitive electromobility shift assays with Cy5-labelled plasmodium nucleosomes (left panel) and Cy3-labelled human nucleosomes (middle panel) reconstituted on 601 DNA. Like in the chromatin remodeling assay, plasmodium and human nucleosomes were mixed at equimolar ratios and incubated with increasing concentrations of SNF2H. However, the electromobility shift reactions were incubated for 10 min at room temperature in the absence of ATP and no competitor DNA was added prior to electrophoresis. The gel was stained by fluorescence imaging (cy5, representing the plasmodium nucleosomes, lanes 1–5; cy3, representing the human nucleososomes, lanes 6–10) and subsequently with ethidium bromide (lanes 11–15). The position of the free DNA and nucleosomes are indicated. (B) Scheme showing the sequence differences of the plasmodium and human histone H4 N-terminus. The RHR box indicates the residues that were previously identified to affect SNF2H ATPase activity. The V to I amino acid change at position 21 is marked by an X. (C) The H4hyb octamers consisting of the plasmodium histones H2A, H2B, H3 and the human histone H4 were reconstituted into nucleosomes and assayed SNF2H dependent nucleosome remodeling (cy5 upper panel) in the presence of equimolar amounts of human nucleosomes (cy3 lower panel). Reactions were incubated for 60 min at 37°C in the presence or absence of ATP, as indicated. Nucleosome positions were analyzed by EMSA and imaged for the Cy5 and Cy3 channel, respectively. The positions of the nucleosomes are indicated on the right side and an asterisk marks a non-specific band. (TIFF) [file ppat.1006080.s005.tiff]

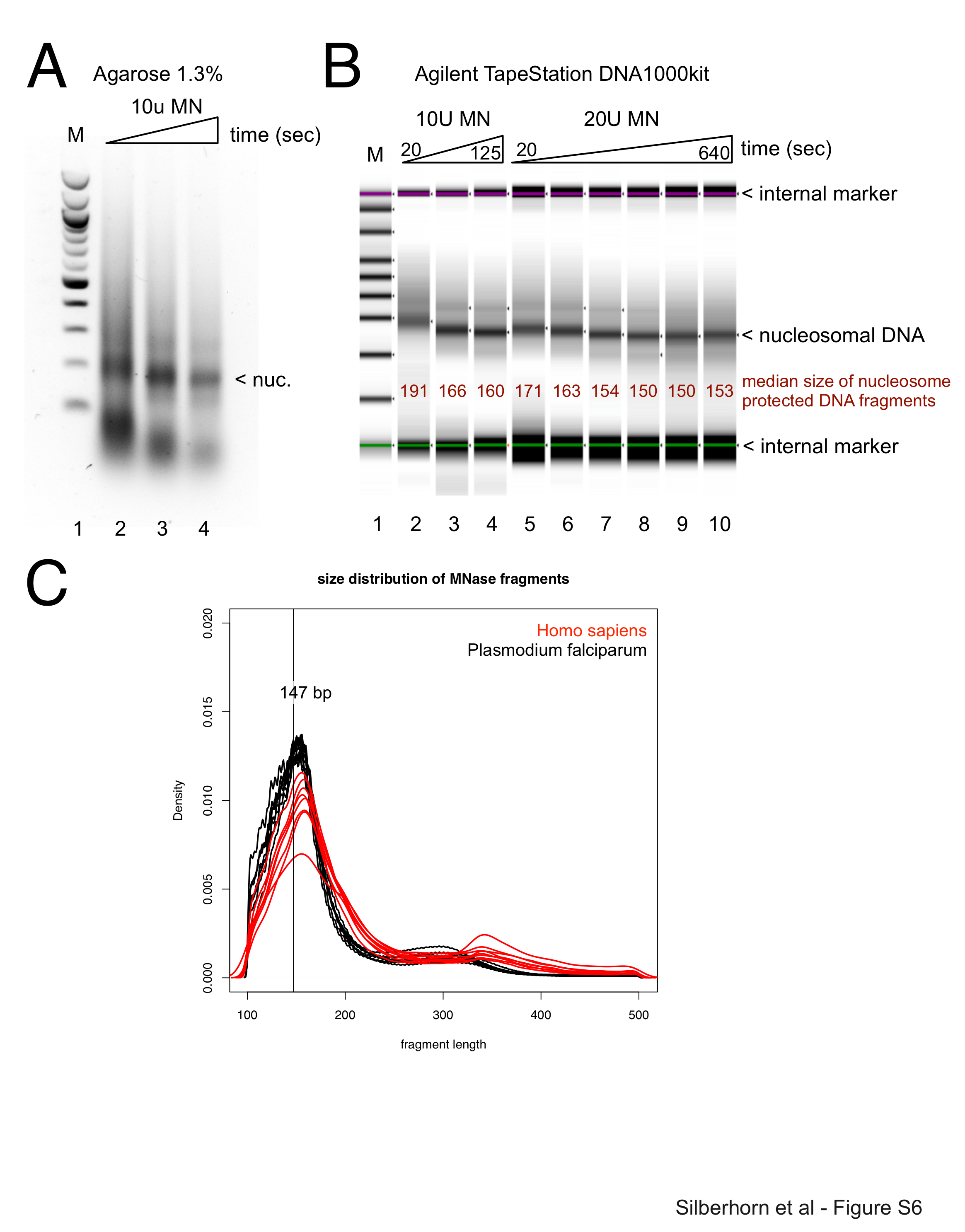

Supplement: S6 Fig — (A) Recombinant plasmodium histone octamers were used for chromatin assembly with a 11kb plasmid by salt dialysis. Chromatin was subjected to partial MNase digestion (10u; 20 to 125 sec), stopping the reaction by the addition of SDS/EDTA. DNA was purified and visualized by agarose gel electrophoresis and ethidium bromide staining. The position of the mono-nucleosomal DNA is indicated (<nuc.). (B) In order to reveal the size of the MNase hydrolysis products in high resolution, samples were separated on the Agilent TapeStation using the DNA1000 kit. Samples shown in (A) (lanes 2–4) and an additional MNase hydrolysis reaction using the twofold amount of MNase (20u) and increased hydrolysis times (20 to 640 sec; lanes 5–10) were analyzed on the TapeStation. The TapeStation analysis software was used to determine the length of the protected DNA fragment and plotted below (median size of nucleosome protected DNA fragment; red). The positions of the protected nucleosomal DNA fragment and the internal system markers are indicated. (C) DNA length distribution plots revealing the MNase protected nucleosomal DNA lengths in vivo. The size distribution of mono-nucleosomal DNA fragments derived from human (red) as well as plasmodium DNA (black) after MNase digestions (SRX885811-SRX885819)[16]. MNase digestions were performed at different stages of the erythrocytic life cycle of P. falciparum. Each stage of the life cycle is plotted individually together with the corresponding human DNA fraction. (TIFF) [file ppat.1006080.s006.tiff]

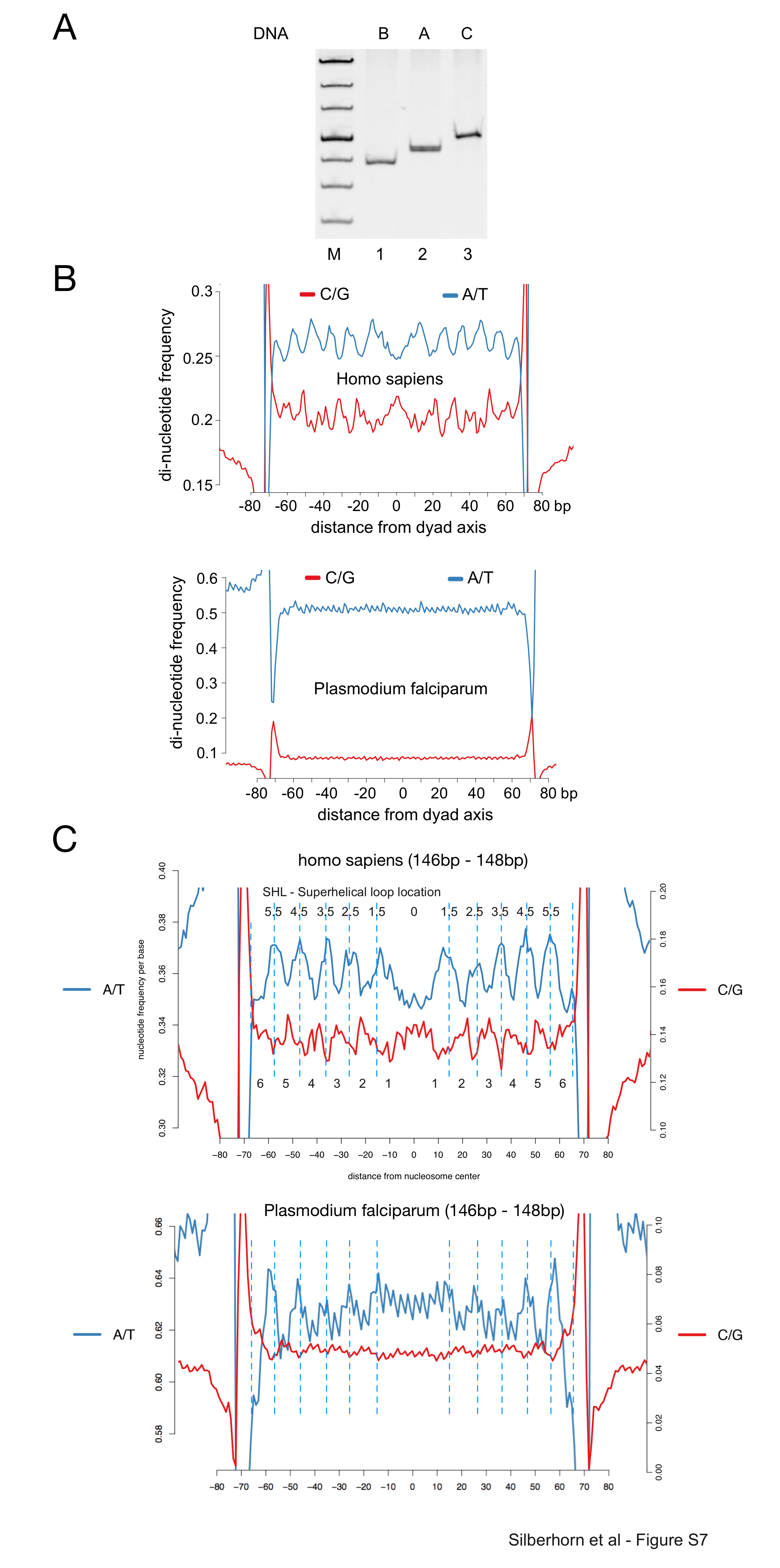

Supplement: S7 Fig — (A) A 6% native gel showing the free DNA used for nucleosome assembly reactions, presented in Fig 5C. The different DNA templates have been prepared by restriction enzyme digestion and purification from a plasmid containing the genomic region from position 1307500–1308399 on chromosome 11. (B) Di-nucleotide frequency of nucleosome core fragments derived from human (left) and plasmodium (right) nucleosomal DNA. Data was extracted from the study performed by the LeRoch laboratories [15]. Position-dependent frequencies of A/T (AA/TT/AT/TA) and C/G (CC/GG/CG/GC) di-nucleotides are illustrated. (C) The same analysis as shown in (B), was performed with the dataset taken from the publication of Kensche and colleagues [16]. Position-dependent frequencies of A/T (AA/TT/AT/TA) and C/G (CC/GG/CG/GC) di-nucleotides are illustrated. The positions of histone-DNA contact points, the superhelical loop locations (SHL), are shown by dotted blue lines and annotated. (TIFF) [file ppat.1006080.s007.tiff]

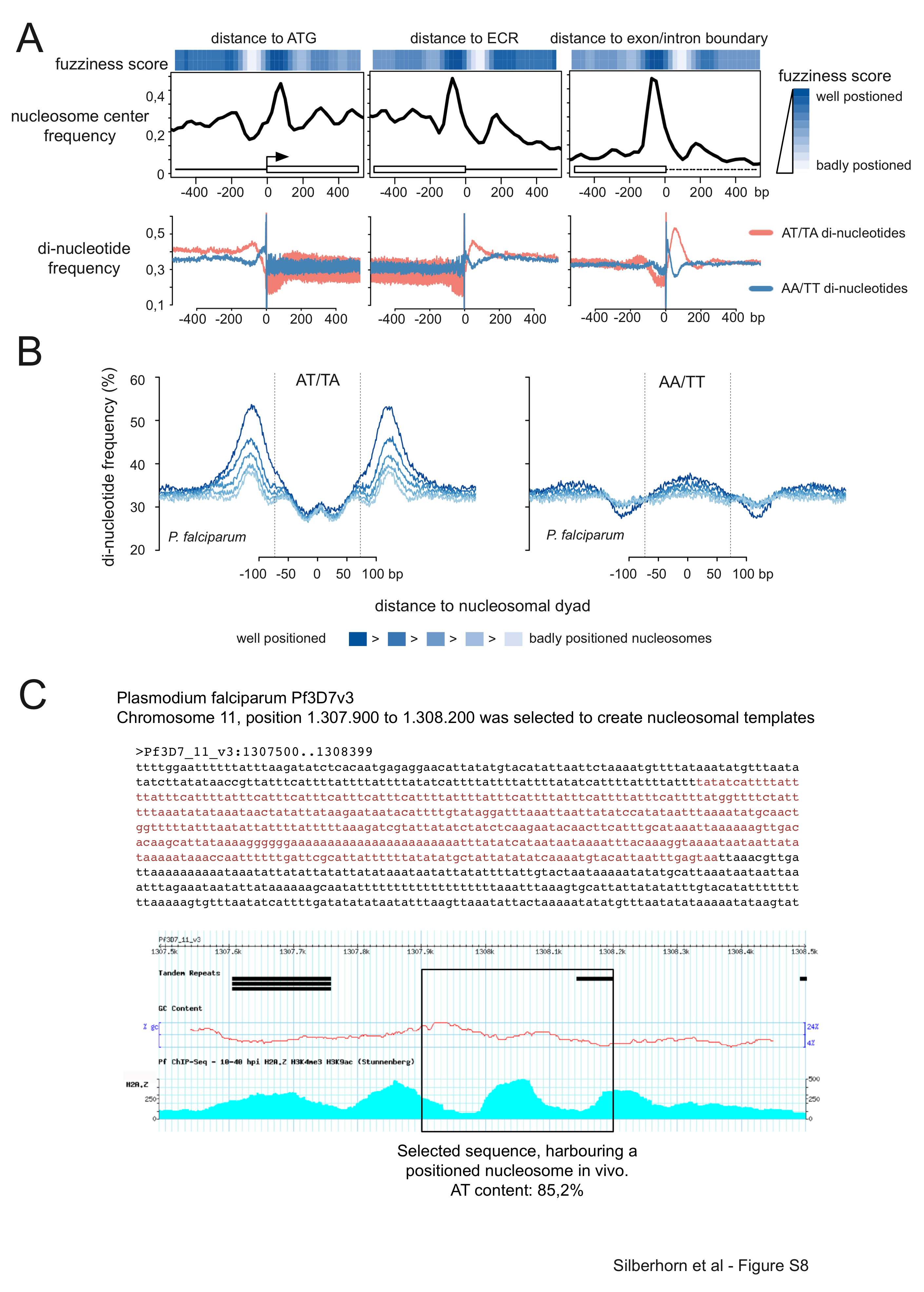

Supplement: S8 Fig — (A) Nucleosome positioning at the beginning of the coding region (ATG), the end of the coding region (ECR) and the exon/intron boundaries of all annotated P. falciparum genes were aligned based on the datasets of Kensche and colleagues [16]. Individual nucleosome positions were further characterized by a fuzziness score and the average fuzziness signal for all aligned genes was plotted as a heatmap (top). The color code is indicated on the right. The average frequency of identified nucleosome midpoints (middle) and of AT/TA (red) and AA/TT di-nucleotides (blue) over regulatory regions of all annotated genes is given. (B) Changes in AT/TA and AA/TT di-nucleotide frequencies within the nucleosome and its linker regions compared to the fuzziness score of nucleosome positioning. Five different fractions of nucleosomes, from well positioned (dark blue) to badly positioned (light blue) nucleosomes for P. falciparum (top panel) and S. cerevisiae (bottom panel) were analyzed. Vertical lines indicate nucleosome boundaries. The color code for the fuzziness score is indicated below. (C) The sequence used to analyze nucleosome positioning in Fig 6C was chosen according to its potential to form positioned nucleosomes in vivo. As a measure for nucleosome positioning we used the ChIP-Seq experiments performed by the Stunnenberg lab [77] that are indicated in the screen shot of the genome browser (lower panel). According to the data, we synthesized a 300bp long DNA fragment, representing the P. falciparum sequence from position 1.307.900 to 1.308.200 of chromosome 11 (Pf3D7 v3). The AT-content of this sequence is 85.2%. (TIFF) [file ppat.1006080.s008.tiff]
